# Identification of MUC1-C as a Target for Suppressing Progression of Head and Neck Squamous Cell Carcinomas

**DOI:** 10.1158/2767-9764.CRC-24-0011

**Published:** 2024-05-14

**Authors:** Ayako Nakashoji, Naoki Haratake, Atrayee Bhattacharya, Weipu Mao, Kangjie Xu, Keyi Wang, Tatsuaki Daimon, Hiroki Ozawa, Keisuke Shigeta, Atsushi Fushimi, Nami Yamashita, Yoshihiro Morimoto, Mototsugu Shimokawa, Shin Saito, Ann Marie Egloff, Ravindra Uppaluri, Mark D. Long, Donald Kufe

**Affiliations:** 1 Dana-Farber Cancer Institute, Harvard Medical School, Boston, Massachusetts.; 2 Central Laboratory Department, Binhai County People's Hospital, Yancheng, P.R. China.; 3 Department of Biostatistics, Graduate School of Medicine, Yamaguchi University, Yamaguchi, Japan.; 4 Department of Biostatistics and Bioinformatics, Roswell Park Comprehensive Cancer Center, Buffalo, New York.

## Abstract

The MUC1-C protein is aberrantly expressed in adenocarcinomas of epithelial barrier tissues and contributes to their progression. Less is known about involvement of MUC1-C in the pathogenesis of squamous cell carcinomas (SCC). Here, we report that the *MUC1* gene is upregulated in advanced head and neck SCCs (HNSCC). Studies of HNSCC cell lines demonstrate that the MUC1-C subunit regulates expression of (i) RIG-I and MDA5 pattern recognition receptors, (ii) STAT1 and IFN regulatory factors, and (iii) downstream IFN-stimulated genes. MUC1-C integrates chronic activation of the STAT1 inflammatory pathway with induction of the *∆Np63* and *SOX2* genes that are aberrantly expressed in HNSCCs. In extending those dependencies, we demonstrate that MUC1-C is necessary for NOTCH3 expression, self-renewal capacity, and tumorigenicity. The findings that MUC1 associates with ∆Np63, SOX2 and NOTCH3 expression by single-cell RNA sequencing analysis further indicate that MUC1-C drives the HNSCC stem cell state and is a target for suppressing HNSCC progression.

**Significance:**

This work reports a previously unrecognized role for MUC1-C in driving STAT1-mediated chronic inflammation with the progression of HNSCC and identifies MUC1-C as a druggable target for advanced HNSCC treatment.

## Introduction

Patients with locally advanced head and neck squamous cell carcinomas (HNSCC) have historically had a poor prognosis with less than 50% surviving for 5 years despite curative-intent treatment with surgery, radiotherapy, and chemotherapy (1–3). Recurrent/metastatic HNSCCs were treated with cisplatin, 5-fluorouracil, and cetuximab, which achieved a response rate of 36% and a progression-free survival of 5.6 months (4). These poor outcomes were substantially improved with development of (i) the immune checkpoint inhibitors (ICI) pembrolizumab and nivolumab as single agents for the second-line treatment of platinum-refractory recurrent/metastatic disease and (ii) pembrolizumab alone or in combination with chemotherapy for first-line treatment (5–7). Those advances for treating HNSCC with immunotherapy fueled trials to evaluate immunochemoradiotherapy and other combined modalities (3). Nonetheless, intrinsic and acquired resistance has remained a challenge for HNSCC immunotherapy as patients invariably succumb to metastatic disease (3). The progression of HNSCC refractory to chemotherapy and ICIs has emphasized a need for the identification of druggable targets to circumvent DNA damage resistance and immune evasion. In this regard, other than cetuximab, there are no approved targeted agents for HNSCC treatment. Potential targets downstream to receptor tyrosine kinases include the transcription factors (TF) (i) ∆Np63 and SOX2 that are dysregulated in HNSCC, and (ii) NOTCH3, which has been linked to stemness and HNSCC progression (8, 9). HNSCCs are associated with chronic exposures to tobacco products and alcohol (8). In addition, human papillomavirus (HPV) infections are linked to an increased incidence of oropharyngeal HNSCCs, in further support of the importance of chronic inflammation in driving HNSCC progression (8–12). Patients with HPV-positive HNSCCs have a more favorable 5-year survival rate than those with HPV-negative HNSCCs and represent a distinct entity in terms of molecular profiles (13–15).

The *mucin 1* (*MUC1*) gene encodes a noncovalent heterodimeric complex consisting of MUC1-N and MUC1-C subunits that evolved in mammals to protect barrier tissues from biotic and abiotic insults (16, 17). As an adverse outcome of this protective function, persistent activation of the transmembrane MUC1-C subunit in settings of chronic inflammation promotes oncogenesis (17–19). In this way, MUC1-C drives lineage plasticity, the epithelial–mesenchymal transition (EMT) and epigenetic reprogramming in the progression of adenocarcinomas derived from barrier glandular epithelia (17–20). Conversely, little is known about the involvement of MUC1-C in SCCs that are derived from stratified squamous epithelial cells (8). Expression of MUC1-N and the MUC1 cytoplasmic tail has been detected in HNSCCs (21–23). Other work has reported that MUC1-N promotes migration and invasion of oral SCC cells (24); however, the mechanisms underlying potential roles for MUC1-C have not been defined in squamous cell biology and HNSCC. In the progression of adenocarcinomas, MUC1-C regulates effectors of chronic inflammation, including (i) pattern recognition receptors (PRR) that induce IFNa/b production, (ii) STAT1 with activation of the type I/II IFN pathways, and (iii) IFN-stimulated genes (ISG) that contribute to DNA damage resistance and immune evasion (18, 20, 25–27). MUC1-C–induced regulation of these inflammatory genes is associated with changes in (i) chromatin accessibility conferred by the SWI/SNF BAF and PBAF chromatin remodeling complexes, and (ii) H3K27ac and H3K4me1/3 levels (18, 20, 25–29). These effects of MUC1-C, which in principle are reversible with restitution of homeostasis, become established in settings of chronic inflammation with progression to the cancer stem cell (CSC) state (17–20).

The barrier role of the head and neck squamous epithelium is disrupted by stress, as for example that induced by exposure to cigarette smoke and alcohol (8). How that loss of homeostasis from chronic inflammation promotes progression to HNSCC remains largely unknown. The current studies demonstrate that MUC1-C drives STAT1-dependent chronic inflammation in HPV-negative HNSCC cells and thereby induction of the ∆Np63 (9, 30–32) and SOX2 (10, 33) TFs that have been linked to HNSCC pathogenesis. We also demonstrate that (i) MUC1-C signaling regulates the NOTCH3 pathway that contributes to the HNSCC CSC state (34, 35), and (ii) HNSCC cells are dependent on MUC1-C for self-renewal and tumorigenicity. In concert with these results, we report that MUC1 associates with ∆Np63, SOX2, and NOTCH3 expression in HNSCC tumor cells as determined by single-cell RNA sequencing (scRNA-seq) analysis. These findings indicate that MUC1-C promotes HNSCC progression and is a potential target for advanced HNSCC treatment.

## Materials and Methods

### Cell Culture

HPV-negative CAL27 cells (ATCC, RRID:CVCL_1107) were cultured in DMEM (Thermo Fisher Scientific) supplemented with 10% FBS (GEMINI Bio-Products). HPV-negative HSC3 and FaDu cells (ATCC, RRID:CVCL_1288 and 1218) were cultured in minimum essential medium (Corning) supplemented with 10% FBS. Authentication of the cells was performed every 3–4 months by short tandem repeat analysis. Cells were monitored for *Mycoplasma* contamination every 3–4 months using the MycoAlert Mycoplasma Detection Kit (Lonza). Cells were maintained for 3 months when performing experiments.

### Gene Silencing

MUC1shRNA#1 (MISSION shRNA TRCN0000122938; Sigma) and a control scrambled shRNA (CshRNA; Sigma) were inserted into the pLKO-tet-puro vector (Plasmid #21915; Addgene) as described previously (36). The MUC1shRNA#2 (MISSION shRNA TRCN0000430218) and STAT1 shRNA (MISSION shRNA TRCN0000004266) were produced in HEK293T cells (RRID:CVCL_0063) as described previously (36). Flag-tagged MUC1-C/CD was inserted into the empty control pLenti CMV Blast DEST (706-1) vector (Plasmid #17451; Addgene) as described previously (27). Vector-transduced cells were selected for growth in 1–2 µg/mL puromycin. Cells were treated with 0.1% DMSO as the vehicle control or 500 ng/mL doxycycline (DOX; Millipore Sigma).

### Immunoblot Analysis

Total lysates prepared from nonconfluent cells were subjected to immunoblot analysis using anti-MUC1-C (HM-1630-P1ABX, 1:200 dilution; Thermo Fisher Scientific), anti-RIG-I [3743S, 1:1,000 dilution; Cell Signaling Technology (CST)], anti-MDA5 (5321S, 1:1,000 dilution; CST), anti-STAT1 (9172S, 1:1,000 dilution; CST), anti-STAT2 (72604S, 1:1,000 dilution; CST), anti-IRF9 (76684S, 1:1,000 dilution; CST), anti-IRF1 (8478S, 1:1,000 dilution; CST), anti-OAS1 (14955-1-AP, 1:1,000 dilution; Proteintech), anti-MX1 (13750-1-AP, 1:1,000 dilution; Proteintech), anti-ISG15 (sc-166755, 1:1,000 dilution; Santa Cruz Biotechnology), anti-GBP1 (15303-1-AP, 1:1,000 dilution; Proteintech), anti-IDO-1 (86630S, 1:1,000 dilution, CST), anti-WARS (GTX110223, 1:1,000 GeneTex), anti-∆Np63 (619002, 1:1,000 dilution; BioLegend), anti-SOX2 (3579, 1:1,000 dilution; CST), anti-NOTCH3 (5276T, 1:1,000 dilution, CST), anti-HEY1 (19929-1-AP, 1:2,000 dilution; Proteintech), anti-JAG1 (2620T, 1:1,000 dilution, CST), anti-DLL3 (2483S, 1:1,000 dilution, CST) and anti-β-actin (A5441, 1:10,000 dilution; Sigma-Aldrich). Chromatin purified using the Chromatin Extraction Kit (ab117152, Abcam) was immunoblotted with anti-MUC1-C (HM-1630-P1ABX, 1:200 dilution; Thermo Fisher Scientific), anti-STAT1 (9172S, 1:1,000 dilution; CST) and anti-histone H3 (ab1791, 1:5,000 dilution, Abcam).

### Colony Formation Assays

Cells were seeded in 24-well plates for 24 hours and then treated with (i) 0.1% DMSO or 500 ng/mL DOX, and (ii) PBS or GO-203. After 7–14 days, cells were stained with 0.5% crystal violet (LabChem) in 25% methanol. Growth was quantified at 590 nm using a spectrophotometer and normalized to DMSO treatment.

### qRT-PCR

Total cellular RNA was isolated using TRIzol reagent (Thermo Fisher Scientific). cDNAs were synthesized using the High Capacity cDNA Reverse Transcription Kit (Applied Biosystems) as described previously (36). The cDNA samples were amplified using the Power SYBR Green PCR Master Mix (Applied Biosystems) and the CFX96 Real-Time PCR System (Bio-Rad) as described previously (36). Primers used for qRT-PCR are listed in Supplementary Table S1.

### Click-iT Nascent RNA Assay

Nascent RNA labeling with ethylene uridine (EU) was performed using the Click-iT Nascent RNA Capture kit (Invitrogen) as described previously (29). Cells were pulsed with 0.5 mmol/L EU for 24 hours and nascent transcripts were captured from isolated total RNA on streptavidin magnetic beads. cDNA synthesis was performed on the beads using the High-Capacity cDNA Reverse Transcription Kit followed by qRT-PCR analysis.

### Chromatin Immunoprecipitation

Chromatin immunoprecipitation (ChIP) was performed on cells cross-linked with 1% formaldehyde for 10 minutes at 37°C, quenched with 2 mol/L glycine, washed with PBS, and sonicated in a Covaris 220 sonicator to generate 300–600 bp DNA fragments. Immunoprecipitation was performed using a control IgG (3900S, CST, RRID:AB_1550038) and antibodies against MUC1-C (16564S, CST, RRID:AB_2798765) and STAT1 (9172S, CST). Quantitation was performed on immunoprecipitated DNA using SYBR-green and the CFX384 real-time PCR machine (Bio-Rad). Precipitated DNAs were detected by PCR using primers listed in Supplementary Table S2. Data are reported as percentage of input DNA for each sample.

### Chromatin-bound Protein Extraction

Chromatin Extraction Kit (Abcam) was used to isolate chromatin-bound proteins according to the manufacturer's instructions.

### RNA-seq Analysis

Total RNA from cells cultured in triplicates was isolated using TRIzol reagent (Invitrogen) as described previously (36). TruSeq Stranded mRNA (Illumina) was used for library preparation. Raw sequencing reads were aligned to the human genome (GRCh38.74) using STAR. Raw feature counts were normalized and differential expression analysis using DESeq2 as described previously (36). Differential expression rank order for subsequent gene set enrichment analysis (GSEA) was performed using the fgsea (v1.8.0) package in R. Hallmark Gene Sets were queried through the Molecular Signatures Database (MSigDB).

### Tumorsphere Formation Assays

Cells (5 × 10^3^) were seeded per well in 6-well ultra-low attachment culture plates (Corning Life Sciences) in DMEM/F12 50/50 medium (Corning Life Sciences) with 20 ng/mL EGF (Millipore Sigma), 20 ng/mL bFGF (Millipore Sigma), and 1% B27 supplement (Gibco). Cells were treated with (i) 0.1% DMSO or 500 ng/mL DOX, and (ii) PBS or GO-203 as described previously (36). Tumorspheres were counted under an inverted microscope in triplicate wells.

### Mouse Tumor Model Studies

Six-week-old nude mice (Jackson Laboratory) were injected subcutaneously into the flank with 1 × 10^7^ CAL27 cells in 100 mL of a 1:1 solution of medium and Matrigel (BD Biosciences). When the mean tumor volume reached 100–150 mm^3^, the mice were pair-matched into groups of 5 mice each. Mice were treated intraperitoneally each day with PBS or GO-203 at a dose of 12 µg/g body weight. Tumor measurements and body weights were recorded twice per week. Tumor lysates were prepared using the T-PER tissue protein extraction reagent (#78510, Thermo Fisher Scientific). The resource equation method was used for determining the minimum number of mice to achieve significance (37). These studies were conducted in accordance with the ethical regulations required for approval by the Dana-Farber Cancer Institute Animal Care and Use Committee under protocol #03–029.

### Analysis of Publicly Available Bulk RNA-seq HNSCC Data

HNSCC RNA-seq and clinical annotation dataset file of GSE136037 was downloaded from the Gene Expression Omnibus (GEO) database. GSE136037 included 49 primary lesions and 23 metastatic lesions. The downloaded data GSE136037 was used in read count format.

### Analysis of Publicly Available scRNA-seq HNSCC Data

Data for publicly available scRNA-seq dataset of HNSCC samples (GSE181919) were obtained from GEO. GSE181919 included 20 primary tumor samples (13 HPV-negative and seven HPV-positive). The expression level of Transcript per million (TPM) was obtained with tumor cells identified using previously determined criteria (38). Uniform manifold approximation and projection (UMAP) representations of remaining tumor cells were produced from total expression profiles, with expression (TPM) compared between given factors. Generated census counts were obtained from GEO. Low-quality cells previously determined were eliminated prior to downstream analysis. Remaining cells were analyzed by Seurat. Normalization and variance stabilization were conducted using regularized negative binomial regression(sctransform).

### Statistical Analysis

Each experiment was performed at least three times. Unpaired two-tailed Student *t* tests were used to assess differences between the mean ± SD of two groups. GraphPad Prism9 was used for all statistical analyses. *P* values were considered significant at *, *P* ≤ 0.05; **, *P* ≤ 0.01; ***, *P* ≤ 0.001; ****, *P* ≤ 0.0001 with confidence interval = 95%.

### Data Availability

Raw and processed RNA-seq data generated in this study are available from the NCBI GEO under accession GSE252287.

## Results

### Dependence of HNSCC Cells on MUC1-C for Clonogenicity

**FIGURE 1:**
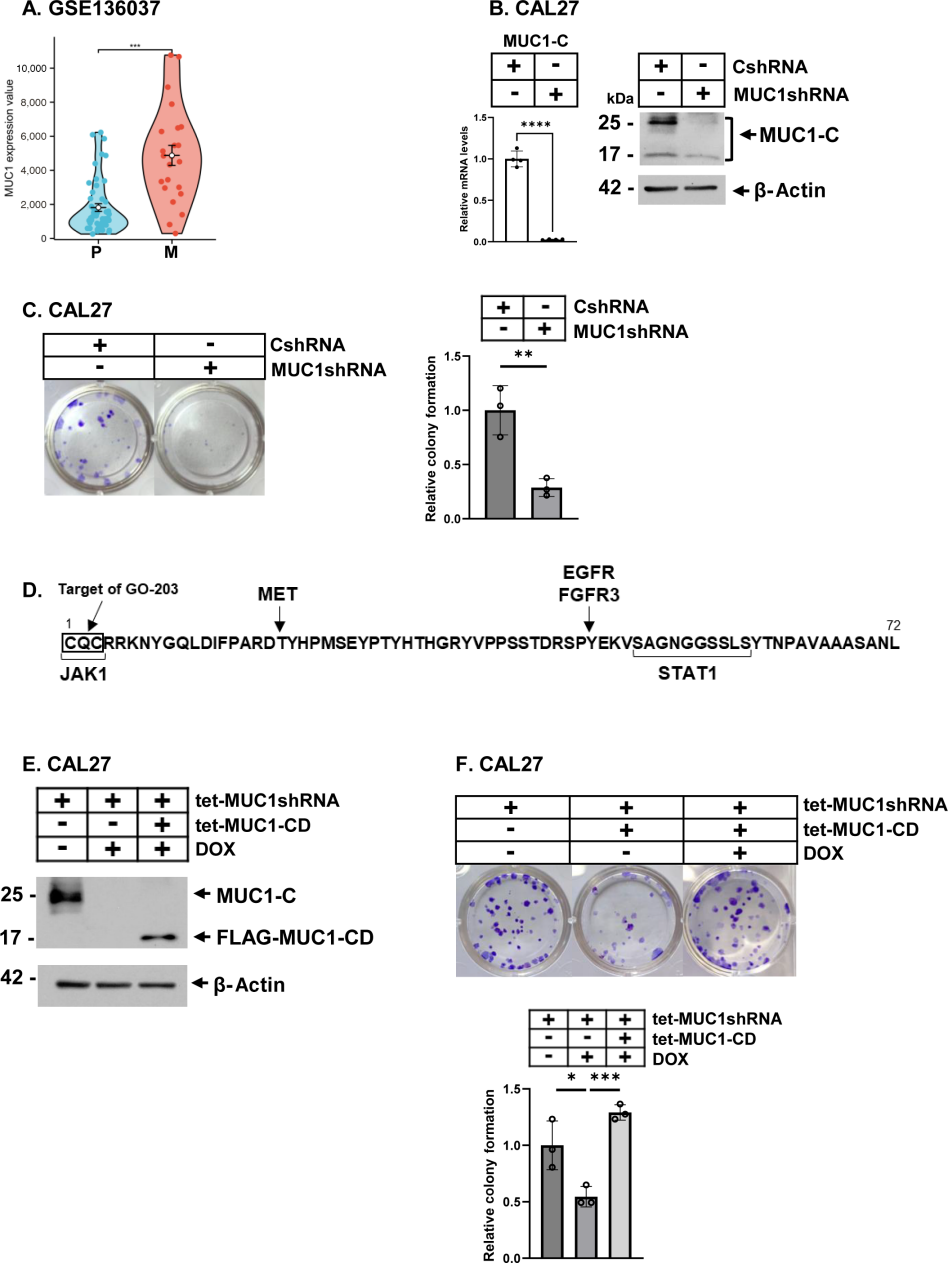
Expression of MUC1 in HNSCC tumors and effects of silencing MUC1-C on HNSCC cell clonogenicity. **A,** Analysis of primary (P) and metastatic (M) HNSCC tissues for MUC1 expression using the GSE136037 dataset. **B,** CAL27/CshRNA and MUC1shRNA cells were analyzed for MUC1-C mRNA levels (left). The results (mean ± SD of four determinations) are expressed as relative levels compared with that obtained for CshRNA cells (assigned a value of 1; left). Lysates were immunoblotted with antibodies against the indicated proteins (right). **C,** The indicated CAL27 cells were analyzed for colony formation. Shown are representative photomicrographs of stained colonies (left). The results (mean ± SD of three determinations) are expressed as relative colony formation compared with that for CshRNA cells (assigned a value of 1; right). **D,** Amino acid sequence of the MUC1-C cytoplasmic domain (CD) highlighting direct interactions with JAK1 and STAT1. **E** and **F,** CAL27 cells expressing the indicated vectors were treated with vehicle or DOX for 7 days. Lysates were immunoblotted with antibodies against the indicated proteins (**E**). Cells were analyzed for colony formation (**F**). Shown are representative photomicrographs of stained colonies (top). The results (mean ± SD of three determinations) are expressed as relative colony formation compared with that for control cells (assigned a value of 1; bottom).

Analysis of the GSE136037 dataset demonstrated that *MUC1* gene expression is significantly increased in metastatic versus primary HNSCC tumors (Fig. 1A; Supplementary Fig. S1A). To investigate potential involvement of MUC1-C in HNSCC, we established HPV-negative CAL27 HNSCC cells expressing a CshRNA or MUC1shRNA vector. MUC1-C mRNA levels were decreased in CAL27/MUC1shRNA, and not CAL27/CshRNA, cells (Fig. 1B, left). Moreover, the MUC1-C protein, which is expressed as glycosylated 25 kDa and unglycosylated 17 kDa forms, was decreased in CAL27/MUC1shRNA cells (Fig. 1B, right). We found that silencing MUC1-C in CAL27/MUC1shRNA cells suppresses their capacity for clonogenic survival (Fig. 1C), in support of the potential importance of this oncoprotein in HNSCC progression. MUC1-C consists of a 58 aa extracellular, 28 aa transmembrane, and 72 aa cytoplasmic domain (18). In addressing how MUC1-C promotes CAL27 cell clonogenicity, the MUC1-C cytoplasmic domain (MUC1-CD) is an intrinsically disordered scaffold with nodes for integrating diverse signaling pathways (Fig. 1D; ref. 18). MUC1-CD is phosphorylated by EGFR, FGFR3, and MET (18). In addition, the MUC1-CD CQC motif, which is targeted by the GO-203 inhibitor, binds directly to JAK1 and the SAGNGGSSLS region associates with STAT1 in facilitating their interactions (Fig. 1D; refs. 18, 39). To further assess the effects of MUC1-C and MUC1-CD, we used an inducible tet-MUC1shRNA targeting a different region, which in response to DOX treatment downregulated MUC1-C expression (Fig. 1E). MUC1-C silencing was rescued by expressing a DOX-inducible Flag-MUC1-CD (tet-Flag-MUC1-CD; Fig. 1E). In this way, we found that MUC1-CD reverses the suppressive effects of silencing MUC1-C on colony formation (Fig. 1F). As an additional control, treatment of CAL27/tet-CshRNA cells with DOX had little if any effect on MUC1-C expression and colony formation (Supplementary Fig. S1B and S1C), indicating that CAL27 cells are dependent on MUC1-CD for clonogenic survival.

### MUC1-C Activates Gene Signatures Associated with Chronic Inflammation

**FIGURE 2:**
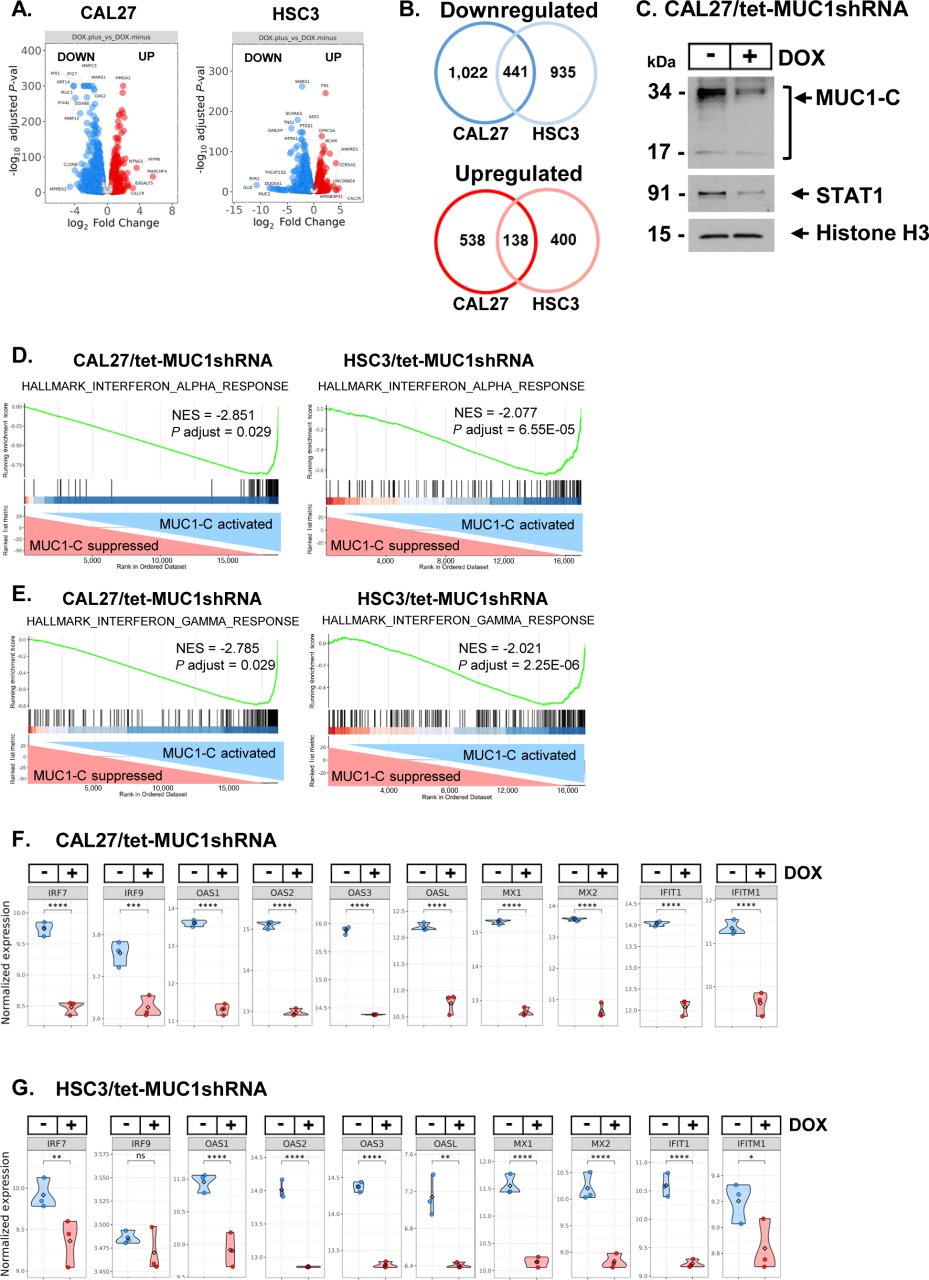
MUC1-C regulates a global transcriptional program enriched for STAT/IRF signaling in HNSCC cells. **A,** RNA-seq was performed on CAL27/tet-MUC1shRNA and HSC3/tet-MUC1shRNA cells treated with vehicle or DOX for 7 days. Volcano plots depicting downregulated (blue) and upregulated (red) DEGs (FDR<0.05; FC>2). Highlighted are the top DEGs by significance and magnitude. **B,** Common downregulated and upregulated genes in CAL27 and HSC3 cells with MUC1-C silencing. **C,** Purified chromatin from CAL27/tet-MUC1shRNA cells treated with vehicle or DOX for 7 days was immunoblotted with antibodies against the indicated proteins. **D** and **E,** Candidate enrichment plots for the HALLMARK INTERFERON ALPHA RESPONSE (**D**), HALLMARK INTERFERON GAMMA RESPONSE (**E**) gene signatures. **F** and **G,** Box plots of selected ISG expression in CAL27 (**F**) and HSC3 (**G**) cells with MUC1-C silencing.

For comparison with CAL27 cells, we established human HPV-negative HSC3 (Supplementary Fig. S2A and S2B) and FaDu (Supplementary Fig. S2C and S2D) HNSCC cells expressing a tet-CshRNA or tet-MUC1shRNA and confirmed that MUC1-C expression is selectively downregulated in DOX-treated tet-MUC1shRNA cells. We also found that, like CAL27 cells, silencing MUC1-C similarly suppresses clonogenicity of HSC3 and FaDu cells (Supplementary Fig. S2E and S2F). Volcano plots demonstrated that inducible MUC1-C silencing in CAL27 and HSC3 cells results in broad changes in downregulated and upregulated gene expression [FDR < 0.05, fold change (FC)>2] (Fig. 2A). By comparing differentially expressed genes (DEG) from CAL27 and HSC3 cells with MUC1-C silencing, we identified (i) 441 common downregulated genes, which included STAT1/2, IRF9, and ISGs, and (ii) 138 upregulated genes (Fig. 2B). MUC1-C localizes to chromatin of cancer cells, where it interacts with TFs and effectors of epigenetic regulation (18, 40). In concert with the findings that MUC1-C binds directly to STAT1 and regulates STAT1 target genes (39), analysis of purified chromatin in CAL27 cells demonstrated that silencing MUC1-C decreases expression of the MUC1-C 17 kDa monomer and 34 kDa homodimer in association with downregulation of STAT1 levels (Fig. 2C). Assessment of the top MUC1-C–downregulated HALLMARK gene sets (Supplementary Fig. S2G) further identified the HALLMARK INTERFERON ALPHA (Fig. 2D) and HALLMARK INTERFERON GAMMA RESPONSE (Fig. 2E) signatures. Consistently, silencing MUC1-C in CAL27 and HSC3 cells was associated with suppression of downstream IFN type I/II pathway ISGs, which included (i) IRF7 and IRF9, (ii) OAS1-3 and OASL, (iii) MX1/2 and (iv) IFIT1 and IFITM1 (Fig. 2F and G).

### MUC1-C Drives Intrinsic Chronic Inflammation by Regulating the Type I/II IFN Pathways

**FIGURE 3:**
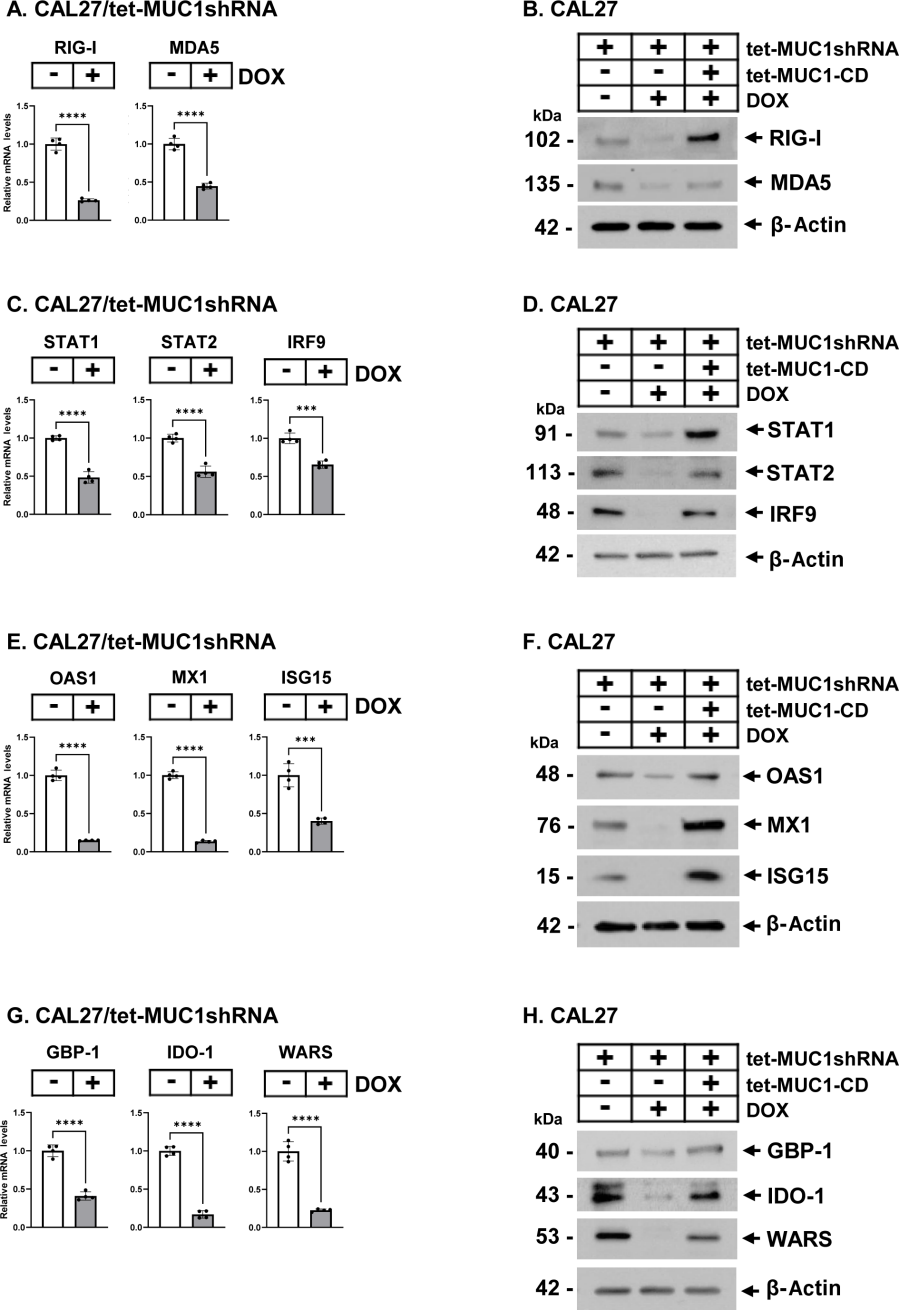
MUC1-C regulates expression of PRRs and effectors of the type I and II IFN pathways. **A,** CAL27/tet-MUC1shRNA cells treated with vehicle or DOX for 6 days were analyzed for RIG-I and MDA5 mRNA levels. The results (mean ± SD of four determinations) are expressed as relative levels compared with that obtained for vehicle-treated cells (assigned a value of 1). **B,** CAL27 cells expressing the indicated vectors were treated with vehicle or DOX for 7 days. Lysates were immunoblotted with antibodies against the indicated proteins. **C,** CAL27/tet-MUC1shRNA cells treated with vehicle or DOX for 7 days were analyzed for STAT1, STAT2, and IRF9 mRNA levels. The results (mean ± SD of four determinations) are expressed as relative levels compared with that obtained for vehicle-treated cells (assigned a value of 1). **D,** CAL27 cells expressing the indicated vectors were treated with vehicle or DOX for 7 days. Lysates were immunoblotted with antibodies against the indicated proteins. **E,** CAL27/tet-MUC1shRNA cells treated with vehicle or DOX for 7 days were analyzed for OAS1, MX1, and ISG15 mRNA levels. The results (mean ± SD of four determinations) are expressed as relative levels compared with that obtained for vehicle-treated cells (assigned a value of 1). **F,** CAL27 cells expressing the indicated vectors were treated with vehicle or DOX for 7 days. Lysates were immunoblotted with antibodies against the indicated proteins. **G,** CAL27/tet-MUC1shRNA cells treated with vehicle or DOX for 7 days were analyzed for GBP-1, IDO-1, and WARS mRNA levels. The results (mean ± SD of four determinations) are expressed as relative levels compared with that obtained for vehicle-treated cells (assigned a value of 1). **H,** CAL27 cells expressing the indicated vectors were treated with vehicle or DOX for 7 days. Lysates were immunoblotted with antibodies against the indicated proteins.

The type I IFN pathway is chronically activated in cancer cells by the RIG-I and MDA5 PRRs that recognize cytosolic RNA (41–43). Silencing MUC1-C in CAL27 cells decreased RIG-I and MDA5 transcripts (Fig. 3A) and protein levels, which were rescued with MUC1-CD (Fig. 3B). Congruent with driving these PRRs, silencing MUC1-C in CAL27 cells suppressed STAT1/2 and IRF9 (Fig. 3C and D). Similar effects on PRR, STAT1/2, and IRF9 expression were observed in HSC3 cells with MUC1-C silencing (Supplementary Fig. S3A and S3B). STAT1/2 regulate expression of downstream ISGs (44). We confirmed that targeting MUC1-C is necessary for expression of OAS1, MX1, and ISG15 proteins that contribute to the IFN-related DNA damage signature (Fig. 3E and F; Supplementary Fig. S3C; refs. 45–48). Among these, OAS1 also amplifies the type I IFN pathway and expression of genes that promote immune evasion (49). In addition, ISG15 links the DNA damage response to regulation of innate immunity (50). Along these lines, we found that MUC1-C regulates expression of guanylate-binding protein 1 (GBP1), which is activated by chronic inflammation and confers treatment resistance (refs. 51, 52; Fig. 3G and H; Supplementary Fig. S3D). Silencing MUC1-C also decreased expression of (i) IDO1, which suppresses tryptophan (Trp) levels in the tumor microenvironment necessary for T-cell function (53), and (ii) tryptophanyl-tRNA synthetase (WARS, WRS) that protects cancer cells from Trp depletion (refs. 54, 55; Fig. 3G and H). Similar results were obtained when targeting MUC1-C in HSC3 cells which, unlike CAL27 cells, have undetectable levels of IDO1 expression (Supplementary Fig. S3D). These findings supported a role for MUC1-C in regulating the type I and II IFN pathways in HNSCC cells and integrating expression of ISGs that promote DNA damage resistance and immune evasion associated with the CSC state (18, 27, 56–58).

### MUC1-C and STAT1 Regulate ∆Np63 Expression

**FIGURE 4:**
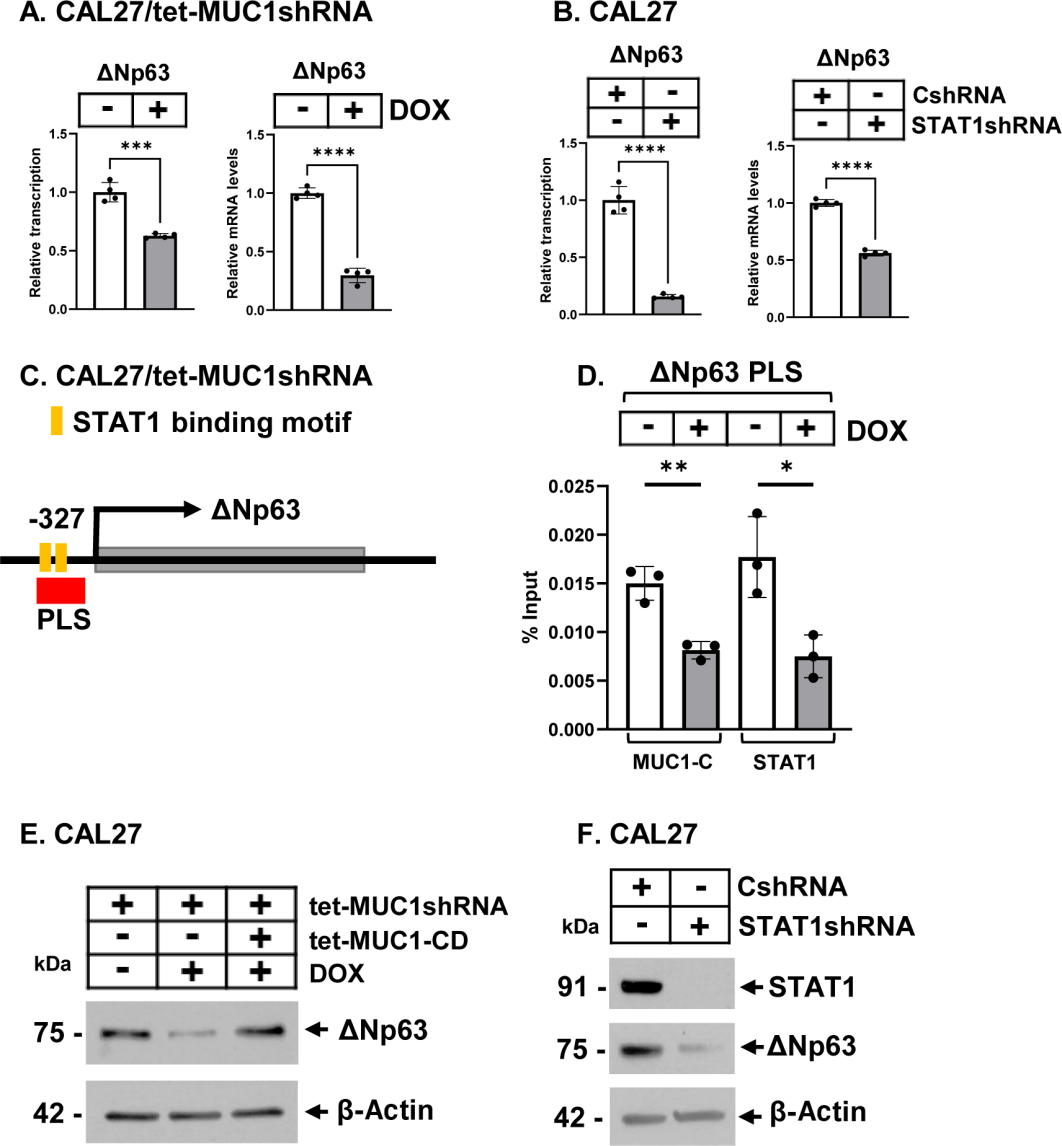
MUC1-C/STAT1 signaling regulates ∆Np63 expression. **A,** CAL27/tet-MUC1shRNA cells treated with vehicle or DOX for 7 days were analyzed for *∆Np63* gene transcription (left) and mRNA (right) levels. The results (mean ± SD of four determinations) are expressed as relative levels compared with that obtained for vehicle-treated cells (assigned a value of 1). **B,** CAL27/CshRNA and CAL27/STAT1shRNA cells were analyzed for *∆Np63* gene transcription (left) and mRNA (right) levels. The results (mean ± SD of four determinations) are expressed as relative levels compared with that obtained for vehicle-treated cells (assigned a value of 1). **C,** Schema of the *∆Np63* gene with highlighting localization of the PLS region that contains potential STAT1 binding motifs. **D,** Soluble chromatin from CAL27/tet-MUC1shRNA cells treated with vehicle or DOX for 7 days was precipitated with anti-MUC1-C and anti-STAT1. The DNA samples were amplified by qPCR with primers for the *∆Np63* PLS region. The results (mean ± SD of three determinations) are expressed as percentage of the input DNA for each sample. **E,** CAL27 cells expressing the indicated vectors were treated with vehicle or DOX for 7 days. Lysates were immunoblotted with antibodies against the indicated proteins. **F,** Lysates from CAL27/CshRNA and CAL27/STAT1shRNA cells were immunoblotted with antibodies against the indicated proteins.

The ∆Np63 TF plays a role in (i) homeostasis of the epidermis, (ii) SCC pathogenesis, and (iii) self-renewal of CSCs (9, 30–32). To our knowledge, there is no known association between MUC1-C and ∆Np63. We found here that targeting MUC1-C decreases *∆Np63* gene transcription and mRNA levels in CAL27 and HSC3 cells (Fig. 4A; Supplementary Fig. S4A). We also found that silencing STAT1 suppresses ∆Np63 expression (Fig. 4B; Supplementary Fig. S4B), supporting potential involvement of the MUC1-C/STAT1 autoinductive pathway (39). *∆Np63* is transcribed from a promoter in the third intron of the *TP63* gene (31), which was of interest in that a promoter-like signature (PLS) in this region contains a potential STAT binding motif (Fig. 4C). In concert with MUC1-C binding to STAT1 in regulating STAT1 target genes (39), ChIP studies of the PLS demonstrated that MUC1-C and STAT1 occupy this region and that silencing MUC1-C decreases their occupancy (Fig. 4D). Consistent with these results, targeting MUC1-C and STAT1 downregulated expression of the ∆Np63 protein (Fig. 4E and F; Supplementary Fig. S4C and S4D), indicating that MUC1-C/STAT1 signaling is necessary for ∆Np63 expression.

### MUC1-C and STAT1 are Necessary for SOX2 Expression

**FIGURE 5:**
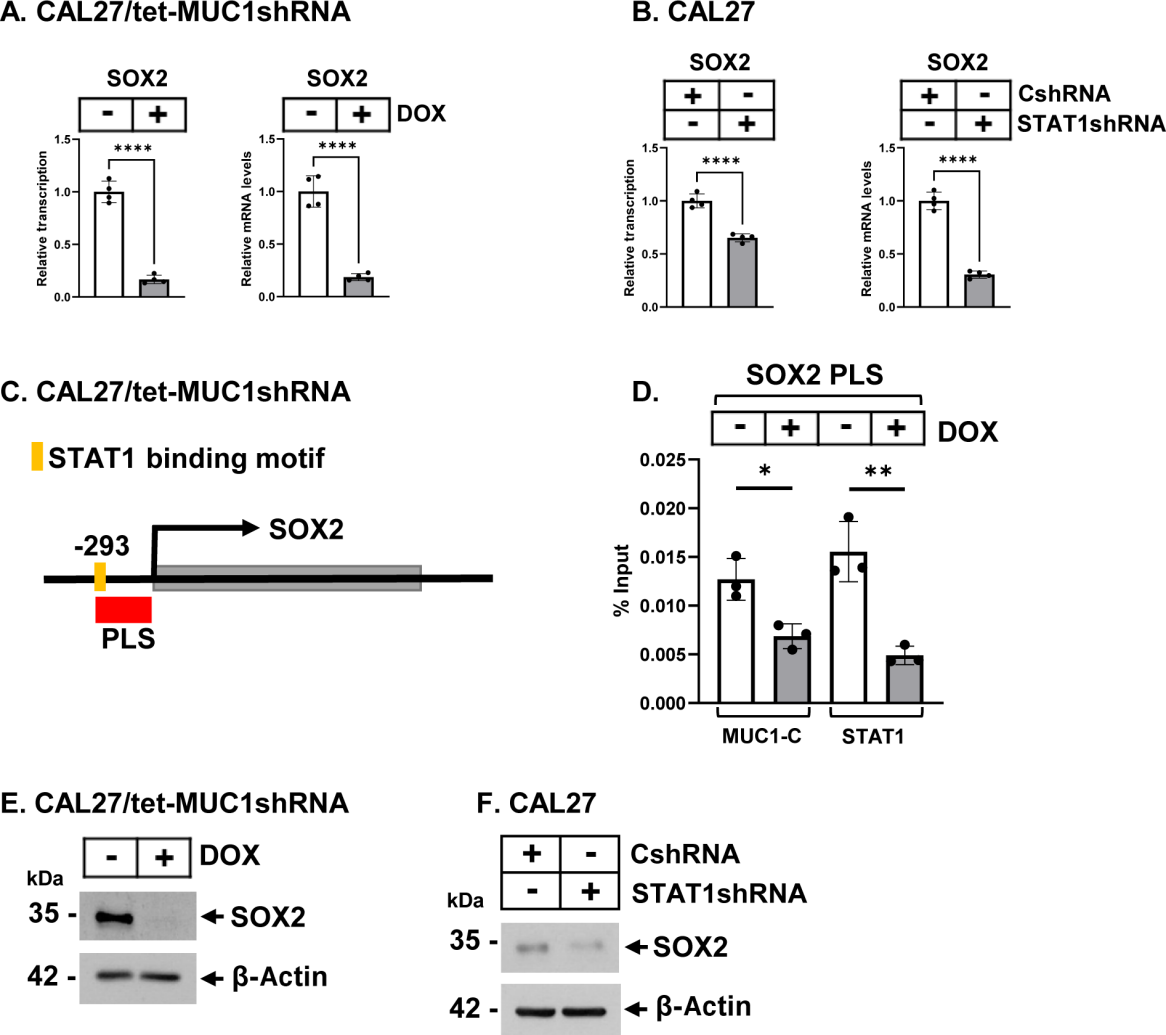
MUC1-C/STAT1 signaling regulates activation of the *SOX2* gene. **A,** CAL27/tet-MUC1shRNA cells treated with vehicle or DOX for 7 days were analyzed for *SOX2* gene transcription (left) and mRNA (right) levels. The results (mean ± SD of four determinations) are expressed as relative levels compared with that obtained for vehicle-treated cells (assigned a value of 1). **B,** CAL27/CshRNA and CAL27/STAT1shRNA cells were analyzed for *SOX2* gene transcription (left) and mRNA (right) levels. The results (mean ± SD of four determinations) are expressed as relative levels compared with that obtained for vehicle-treated cells (assigned a value of 1). **C,** Schema of the *SOX2* gene with highlighting localization of the PLS region that contains STAT1 binding motifs. **D,** Soluble chromatin from CAL27/tet-MUC1shRNA cells treated with vehicle or DOX for 7 days was precipitated with anti-MUC1-C and anti-STAT1. The DNA samples were amplified by qPCR with primers for the *SOX2* PLS region. The results (mean ± SD of three determinations) are expressed as percentage of input DNA for each sample. **E,** Lysates from CAL27/tet-MUC1shRNA cells treated with vehicle or DOX for 7 days were immunoblotted with antibodies against the indicated proteins. **F,** Lysates from CAL27/CshRNA and CAL27/STAT1shRNA cells were immunoblotted with antibodies against the indicated proteins.

SOX2 is essential for self-renewal of basal cells, which are the proposed origin of SCCs (30, 59). In addition, SOX2 contributes to progression of the SCC CSC state (10, 33). As found for ∆Np63, targeting MUC1-C in CAL27 and HSC3 cells downregulated *SOX2* gene transcription and mRNA levels (Fig. 5A; Supplementary Fig. S5A). Silencing STAT1 also decreased activation of the *SOX2* gene (Fig. 5B; Supplementary Fig. S5B), indicating that MUC1-C/STAT1 complexes drive SOX2 expression. In addressing this notion, we identified STAT1 binding motifs in a *SOX2* PLS (Fig. 5C) that were occupied by STAT1 in a MUC1-C–dependent manner (Fig. 5D). Targeting MUC1-C and STAT1 also decreased expression of the SOX2 protein (Fig. 5E and F; Supplementary Fig. S5C and S5D), indicating that MUC1-C/STAT1 signaling integrates regulation of the *∆Np63* and *SOX2* genes in HNSCC cells.

### Targeting MUC1-C Downregulates NOTCH3 and HNSCC Cell Self-Renewal Capacity

**FIGURE 6:**
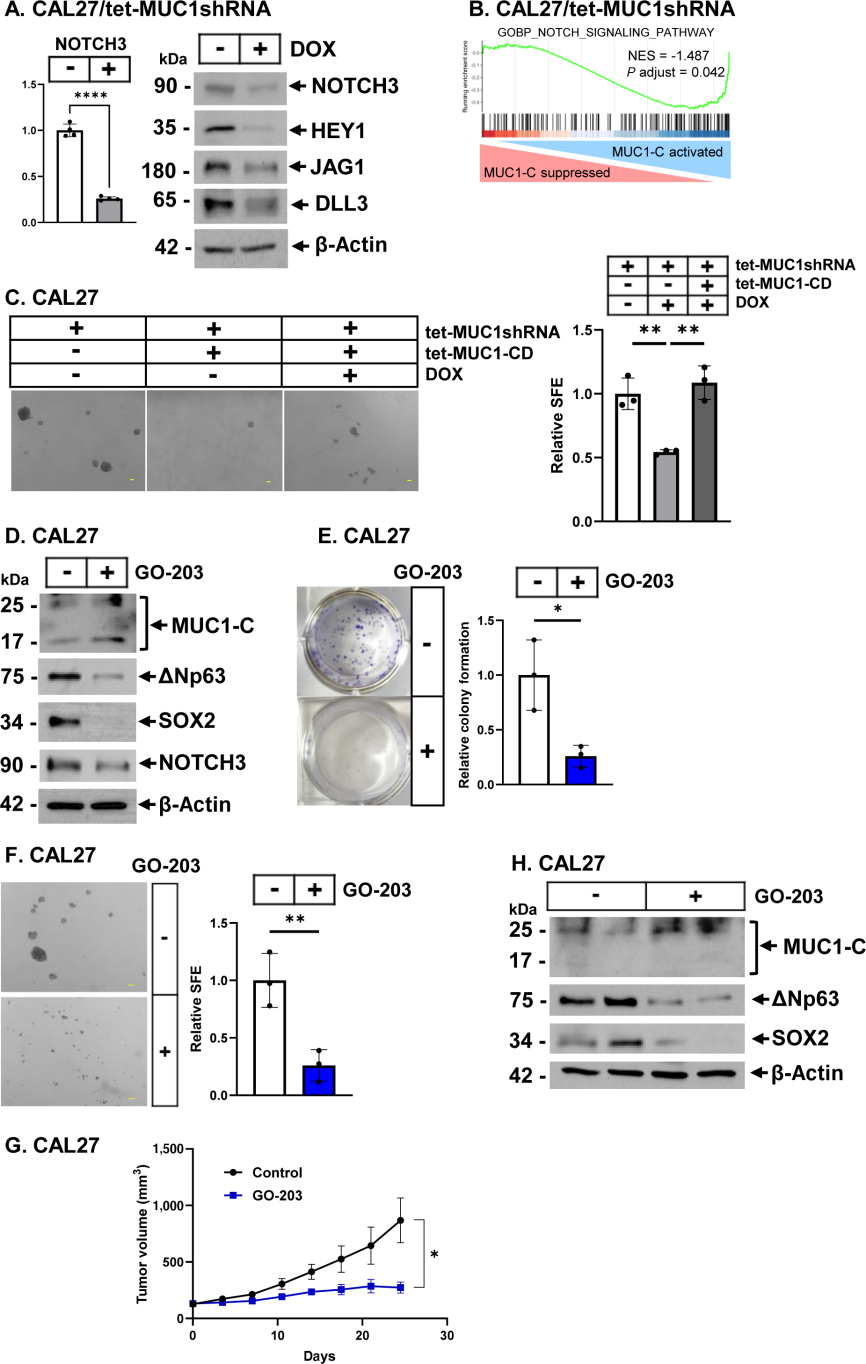
MUC1-C regulates NOTCH3 expression and HNSCC cell self-renewal capacity. **A,** CAL27/tet-MUC1shRNA cells treated with vehicle or DOX for 7 days were analyzed for NOTCH3 mRNA levels. The results (mean ± SD of four determinations) are expressed as relative levels compared with that obtained for vehicle-treated cells (assigned a value of 1; left). Lysates were immunoblotted with antibodies against the indicated proteins (right). **B,** GSEA of RNA-seq data from CAL27/tet-MUC1shRNA cells treated with vehicle or DOX for 7 days using the REACTOME SIGNALING BY NOTCH3 gene signature. **C,** The indicated CAL27 cells treated with vehicle or DOX for 7 days were analyzed for tumorsphere formation. Shown are representative images of the tumorspheres (bar represents 100 µm). SFE is expressed as the mean ± SD of three independent replicates relative to that obtained for vehicle-treated cells (assigned a value of 1). **D,** Lysates from CAL27 cells treated with vehicle or 5 µmol/L GO-203 for 3 days were immunoblotted with antibodies against the indicated proteins. **E,** CAL27 cells were treated with vehicle or 5 µmol/L GO-203 while they were analyzed for colony formation. Shown are representative photomicrographs of stained colonies (left). The results (mean ± SD of three determinations) are expressed as relative colony formation compared with that for vehicle-treated cells (assigned a value of 1; right). **F,** CAL27 cells were treated with vehicle or 5 µmol/L GO-203 while they were analyzed for tumorsphere formation. Shown are representative images of the tumorspheres (bar represents 100 µm). SFE is expressed as the mean ± SD of three independent replicates relative to that obtained for untreated cells (assigned a value of 1). **G** and **H,** Six-week-old nude mice were injected subcutaneously in the flank with 1 × 10^7^ CAL27 cells. Mice pair-matched into groups of 5 mice each when tumors reached 100–150 mm^3^ were treated with vehicle control or GO-203 for the indicated days. Tumor volumes are expressed as the mean ± SEM for 5 mice (**G**). Lysates from control and GO-203–treated tumors were immunoblotted with antibodies against the indicated proteins (**H**).

Functional and genomic analyses have identified an important role for the NOTCH3 stemness factor in HNSCC progression (35). Silencing MUC1-C in CAL27 and HSC3 cells decreased expression of (i) NOTCH3, (ii) jagged canonical ligand 1 (JAG1), (iii) the delta-like ligand 3 (DLL3), and (iv) downstream NOTCH target HEY1 (refs. 35, 60; Fig. 6A; Supplementary Fig. S6A). Concordant with these results, silencing MUC1-C suppressed the GOBP NOTCH SIGNALING PATHWAY and REACTOME SIGNALING BY NOTCH gene signatures (Fig. 6B; Supplementary Fig. S6B). Dysregulation of ∆Np63, SOX2, and NOTCH3 have each been linked to HNSCC cell stemness (8–10, 30–32, 34, 35). Consistent with MUC1-C dependence for ∆Np63, SOX2, and NOTCH3 expression, silencing MUC1-C in CAL27 and HSC3 cells decreased self-renewal capacity as evidenced by suppression of tumorsphere formation (Fig. 6C; Supplementary Fig. S6C). Moreover, we confirmed that MUC1-CD recovers sphere forming efficiency (SFE; Fig. 6C; Supplementary Fig. S6C). The 72 aa MUC1-C cytoplasmic domain includes a CQC motif that is essential for MUC1-C dimerization and function (Fig. 1C; refs. 17, 18). Targeting the MUC1-C CQC motif with the GO-203 inhibitor, which blocks MUC1-C nuclear localization and function (17, 18) decreased ∆Np63, SOX2, and NOTCH3 expression (Fig. 6D; Supplementary Fig. S6D). GO-203 treatment of CAL27 (Fig. 6E) and HSC3 (Supplementary Fig. S6E) cells also suppressed colony formation, confirming dependence of HNSCC cells on MUC1-C for clonogenic survival. In addition, targeting MUC1-C with GO-203 significantly inhibited SFE of CAL27 (Fig. 6F) and HSC3 (Supplementary Fig. S6F) cells, demonstrating that MUC1-C is necessary for their self-renewal capacity. GO-203 treatment of mice with established CAL27 xenografts further demonstrated dependence on MUC1-C for tumorigenicity (Fig. 6G). Immunoblot analysis of CAL27 tumors from control and GO-203–treated mice confirmed that targeting MUC1-C downregulates ∆Np63 and SOX2 expression (Fig. 6H).

### MUC1 Expression in HNSCC Tissues

**FIGURE 7:**
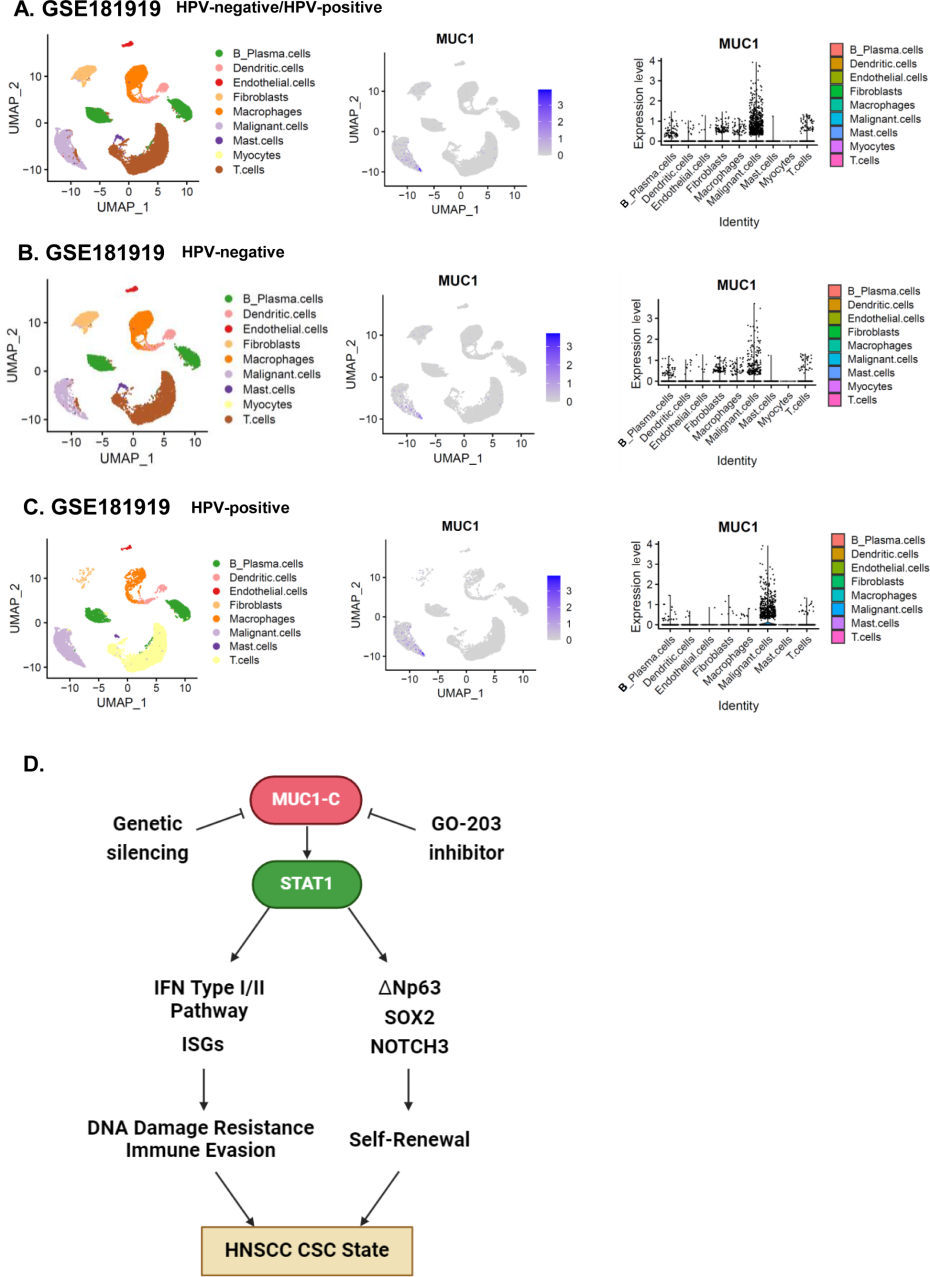
Single-cell profiling of the expression of MUC1 and related genes in HNSCC. **A–C,** UMAP representation of total cells analyzed from the GSE118389 dataset of HPV-negative/HPV-positive HNSCC samples (**A**), GSE118389 dataset of HPV-negative samples (**B**), and GSE118389 dataset of HPV-positive samples (**C**) (left). Distributions of MUC1 expression in individual cells (middle). MUC1 expression in individual cells in each cell type (right). **D,** Model depicting the current findings that MUC1-C integrates activation of STAT1, IFN type I/II signaling and ISG expression with regulation of ∆Np63, SOX2, and NOTCH3 in driving the HNSCC CSC state that promotes DNA damage resistance and immune evasion.

To extend our findings to HNSCC tumors, we analyzed the GSE181919 scRNA-seq dataset derived from 20 primary HNSCCs and four HNSCCs metastatic to lymph nodes (38). HNSCC cells were distinguished from nonmalignant cell types by the presence of DNA copy-number aberrations (CNA; refs. 38, 61). Analysis of malignant HNSCC cells with recurrent CNAs demonstrated higher levels of MUC1 expression compared with that in other cell types (Fig. 7A). The GSE181919 dataset includes HPV-negative and HPV-positive HNSCCs (38), which differ in DNA methylation and gene expression profiles (15). CAL27 and HSC3 cells studied here are HPV-negative; accordingly, we analyzed HPV-negative HNSCCs in the GSE181919 dataset and found similar upregulation of MUC1 expression in the malignant cell population (Fig. 7B). Analysis of the HPV-negative/HPV-positive (Supplementary Fig. S7A) and HPV-negative (Fig. 7C) HNSCC data also detected expression of STAT1, ∆Np63, SOX2, and NOTCH3 in HNSCC cells and, to a variable extent, in other cell populations. Further analysis of malignant HNSCC cells in the HPV-negative/HPV-positive (Supplementary Fig. S7B) and HPV-negative (Supplementary Fig. S7C) datasets uncovered significant correlations of MUC1 expression with ∆Np63, SOX2, and NOTCH3. By contrast, correlations of MUC1 with STAT1 were not significant in terms of their transcripts, which may be related to the findings that MUC1-C largely regulates STAT1 function by direct interactions at the posttranscriptional level (39). Of further interest, analysis of HPV-positive HNSCC tumors demonstrated MUC1 expression in the malignant cell population (Supplementary Fig. S7D), providing preliminary evidence for subsequent studies to determine if MUC1-C also plays a role in the progression of HPV-positive HNSCCs.

## Discussion

The *MUC1* gene evolved in mammals to protect barrier tissues from loss of homeostasis by inflammatory insults (17, 18, 62). The MUC1-C subunit activates EMT, epigenetic reprogramming, and repair responses to stress that, if prolonged as in settings of chronic inflammation, contribute to cancer progression (17, 18, 62). MUC1-C has been largely associated with adenocarcinomas that arise from barrier glandular epithelial cells lining internal organs (17, 18, 62). The current findings demonstrate that *MUC1* is upregulated in metastatic HNSCCs that are derived from barrier stratified squamous epithelial cells (8). Advanced HNSCCs invariably become refractory to multimodality treatment with chemotherapy, radiotherapy, and immunotherapy in association with development of DNA damage resistance and immune evasion (3, 63, 64). In regard to novelty, the current studies uncover involvement of MUC1-C in contributing to HNSCC cell intrinsic pathways of chronic inflammation. In this way, MUC1-C regulates expression of the RIG-I and MDA5 PRRs that are activated by cytosolic RNA (41–43). MUC1-C promotes DNA replicative stress; however, a role in increasing cytosolic RNA is not known (17, 18, 62). Nonetheless, RIG-I and MDA5 contribute to intrinsic upregulation of IFNb production and the type I IFN pathway (27, 65, 66). MUC1-C binds to STAT1 and regulates transactivation of STAT1 target genes (39). In concert with this function and the role of STAT1 in activation of the type I/II IFN pathways, we found that MUC1-C regulates intrinsic activation of downstream ISGs, including OAS1, MX1, and ISG15 that are effectors of DNA damage resistance and immune evasion (Fig. 7D; refs. 41, 47, 49).

Barrier tissues are dependent on resident stem cells (SC) that, following injury, are activated to promote wound healing (67). SC lineage infidelity occurs transiently in wound healing, but persists in settings of chronic inflammation that promote cancer progression (68). Our results uncover the previously unrecognized findings that MUC1-C–induced chronic activation of the STAT1 inflammatory pathway in HNSCC cells regulates the lineage-dictating *∆Np63* and *SOX2* genes, which are amplified in HNSCCs and contribute to HNSCC pathogenesis (Fig. 7D; refs. 8, 69). Mechanistically, we found that MUC1-C and STAT1 occupy the *∆Np63* gene and that silencing MUC1-C decreases STAT1 occupancy and ∆Np63 expression. *∆Np63* is coamplified with *SOX2* in SCCs and the ∆Np63 and SOX2 proteins form a direct complex that regulates SCC gene expression (8, 69). Like *∆Np63*, we found that the *SOX2* gene is occupied by MUC1-C and STAT1. MUC1-C was also necessary for STAT1 occupancy of *SOX2* and targeting MUC1-C and STAT1 downregulated SOX2 expression. These unanticipated results support a model in which MUC1-C/STAT1 complexes regulate ∆Np63 and SOX2, which are necessary for driving the HNSCC CSC state (Fig. 7D; refs. 8, 9, 30–32, 70). By extension and in addition to ∆Np63 and SOX2, we found that MUC1-C regulates expression of NOTCH3, which is necessary for HNSCC cell self-renewal and progression (35).

∆Np63, SOX2, and NOTCH3 have each been shown to be necessary for conferring the HNSCC CSC state (8, 9, 30–32, 70). To our knowledge, there is no evidence for a common pathway that integrates these TFs in driving HNSCC pathogenesis. Our results support a model in which MUC1-C regulates intrinsic chronic inflammation in HNSCC cells through activation of STAT1 and the IFN type I/II pathways (Fig. 7D). In this way, targeting MUC1-C genetically and pharmacologically with the GO-203 inhibitor regulates downstream ISGs that contribute to DNA damage resistance and immune evasion (Fig. 7D). Our results also support a previously unreported role for MUC1-C/STAT1 signaling in integrating chronic inflammation with regulation of (i) lineage-dictating ∆Np63 and SOX2 TFs, and (ii) NOTCH3 and self-renewal capacity (Fig. 7D). Collectively, these findings and the demonstration that MUC1 associates with expression of ∆Np63, SOX2, and NOTCH3 in individual HNSCC tumor cells indicate that MUC1-C is a common effector of the HNSCC CSC state (Fig. 7D). Chronic inflammation is an established driver of cancer progression and therapeutic resistance, albeit by unifying mechanisms that have remained unclear (71). MUC1-C is activated in barrier tissues in response to inflammation (18). Squamous epithelia of the head and neck are exposed to cigarette smoke and alcohol that contribute to chronic inflammation (8). In settings of chronic inflammation with repetitive cycles of damage and repair, prolonged MUC1-C activation becomes established in promoting cancer progression (18). The present findings shed light on the potential involvement of MUC1-C activation in chronic inflammation of squamous epithelia that extends to driving progression of the HNSCC CSC state (Fig. 7D). In addition, our findings support MUC1-C as a target for the treatment of HNSCCs with anti-MUC1-C CAR T cells and antibody–drug conjugates that are under clinical and preclinical development.

## Supplementary Material

Figure S1 Effects of silencing MUC1-C on HNSCC cell clonogenic survival.

Figure S2 MUC1-C regulates the type I and IFN pathways in CAL27 and HSC3 cells.

Figure S3 Effects of targeting MUC1-C on effectors of the type I and II IFN pathways.

Figure S4 Regulation of ∆Np63 expression in HSC3 cells.

Figure S5 Regulation of SOX2 expression in HSC3 cells.

Figure S6 Effects of targeting MUC1-C with the GO-203 inhibitor.

Figure S7 Single-cell profiling of the expression of MUC1 and related genes in HNSCC.

Table S1 Primers used for qRT-PCR analysis.

Table S2 Primers used for ChIP-PCR.

## References

[1] Lacas B , Carmel A, Landais C, Wong SJ, Licitra L, Tobias JS, . Meta-analysis of chemotherapy in head and neck cancer (MACH-NC): an update on 107 randomized trials and 19,805 patients, on behalf of MACH-NC Group. Radiother Oncol 2021;156:281–93. 33515668 10.1016/j.radonc.2021.01.013 PMC8386522

[2] Muzaffar J , Bari S, Kirtane K, Chung CH. Recent advances and future directions in clinical management of head and neck cquamous cell carcinoma. Cancers 2021;13;338. 33477635 10.3390/cancers13020338 PMC7831487

[3] Rao YJ , Goodman JF, Haroun F, Bauman JE. Integrating immunotherapy into multimodal treatment of head and neck cancer. Cancers 2023;15:672. 36765627 10.3390/cancers15030672 PMC9913370

[4] Vermorken JB , Mesia R, Rivera F, Remenar E, Kawecki A, Rottey S, . Platinum-based chemotherapy plus cetuximab in head and neck cancer. N Engl J Med 2008;359:1116–27. 18784101 10.1056/NEJMoa0802656

[5] Ferris RL , Blumenschein G Jr, Fayette J, Guigay J, Colevas AD, Licitra L, . Nivolumab for recurrent squamous-cell carcinoma of the head and neck. N Engl J Med 2016;375:1856–67. 27718784 10.1056/NEJMoa1602252 PMC5564292

[6] Burtness B , Harrington KJ, Greil R, Soulieres D, Tahara M, de Castro G Jr, . Pembrolizumab alone or with chemotherapy versus cetuximab with chemotherapy for recurrent or metastatic squamous cell carcinoma of the head and neck (KEYNOTE-048): a randomised, open-label, phase 3 study. Lancet 2019;394:1915–28. 31679945 10.1016/S0140-6736(19)32591-7

[7] Cohen EEW , Soulieres D, Le Tourneau C, Dinis J, Licitra L, Ahn MJ, . Pembrolizumab versus methotrexate, docetaxel, or cetuximab for recurrent or metastatic head-and-neck squamous cell carcinoma (KEYNOTE-040): a randomised, open-label, phase 3 study. Lancet 2019;393:156–67. 30509740 10.1016/S0140-6736(18)31999-8

[8] Dotto GP , Rustgi AK. Squamous cell cancers: a unified perspective on biology and genetics. Cancer Cell 2016;29:622–37. 27165741 10.1016/j.ccell.2016.04.004 PMC4870309

[9] Gatti V , Fierro C, Annicchiarico-Petruzzelli M, Melino G, Peschiaroli A. DeltaNp63 in squamous cell carcinoma: defining the oncogenic routes affecting epigenetic landscape and tumour microenvironment. Mol Oncol 2019;13:981–1001. 30845357 10.1002/1878-0261.12473 PMC6487733

[10] Singh P , Augustine D, Rao RS, Patil S, Awan KH, Sowmya SV, . Role of cancer stem cells in head-and-neck squamous cell carcinoma – a systematic review. J Carcinog 2021;20:12. 34729044 10.4103/jcar.JCar_14_20 PMC8511833

[11] Salem A , Salo T. Identity matters: cancer stem cells and tumour plasticity in head and neck squamous cell carcinoma. Expert Rev Mol Med 2023;25:e8. 36740973 10.1017/erm.2023.4

[12] McKeon MG , Gallant JN, Kim YJ, Das SR. It takes two to tango: a review of oncogenic virus and host microbiome associated Inflammation in head and neck cancer. Cancers 2022;14:3120. 35804891 10.3390/cancers14133120 PMC9265087

[13] Johnson DE , Burtness B, Leemans CR, Lui VWY, Bauman JE, Grandis JR. Head and neck squamous cell carcinoma. Nat Rev Dis Primers 2020;6:92. 33243986 10.1038/s41572-020-00224-3 PMC7944998

[14] Sun Y , Wang Z, Qiu S, Wang R. Therapeutic strategies of different HPV status in head and neck squamous cell carcinoma. Int J Biol Sci 2021;17:1104–18. 33867833 10.7150/ijbs.58077 PMC8040311

[15] Burkitt K . Role of DNA methylation profiles as potential biomarkers and novel therapeutic targets in head and neck cancer. Cancers 2023;15:4685. 37835379 10.3390/cancers15194685 PMC10571524

[16] Kufe D . Mucins in cancer: function, prognosis and therapy. Nat Rev Cancer 2009;9:874–85. 19935676 10.1038/nrc2761 PMC2951677

[17] Kufe D . MUC1-C in chronic inflammation and carcinogenesis; emergence as a target for cancer treatment. Carcinogenesis 2020;41:1173–83. 32710608 10.1093/carcin/bgaa082 PMC7513951

[18] Kufe D . Emergence of MUC1 in mammals for adaptation of barrier epithelia Cancers 2022;14:4805. 36230728 10.3390/cancers14194805 PMC9564314

[19] Kufe D . Dependence on MUC1-C in progression of neuroendocrine prostate cancer. Int J Mol Sci 2023;24:3719. 36835130 10.3390/ijms24043719 PMC9967814

[20] Yamashita N , Kufe D. Addiction of cancer stem cells to MUC1-C in triple-negative breast cancer progression Int J Mol Sci 2022;23:8219. 35897789 10.3390/ijms23158219 PMC9331006

[21] Rabassa ME , Croce MV, Pereyra A., Segal-Eiras A. MUC1 expression and anti-MUC1 serum immune response in head and neck squamous cell carcinoma (HNSCC): a multivariate analysis. BMC Cancer 2006;6:253. 17064405 10.1186/1471-2407-6-253 PMC1633744

[22] Sipaul F , Birchall M, Corfield A. What role do mucins have in the development of laryngeal squamous cell carcinoma? A systematic review. Eur Arch Otorhinolaryngol 2011;268:1109–17. 21526360 10.1007/s00405-011-1617-8

[23] Boldrup L , Coates P, Gu X, Wang L, Fahraeus R, Wilms T, . Levels of MUC1 in tumours and serum of patients with different sub-types of squamous cell carcinoma of the head and neck. Oncol Lett 2020;20:1709–18. 32724413 10.3892/ol.2020.11746 PMC7377060

[24] Li P , Wu F, Zhao H, Dou L, Wang Y, Guo C, . Analysis of the factors affecting lymph node metastasis and the prognosis of rectal neuroendocrine tumors. Int J Clin Exp Pathol 2015;8:13331–8. 26722537 PMC4680482

[25] Hagiwara M , Yasumizu Y, Yamashita N, Rajabi H, Fushimi A, Long MD, . MUC1-C activates the BAF (mSWI/SNF) complex in prostate cancer stem cells. Cancer Res 2021;81:1111–22. 33323379 10.1158/0008-5472.CAN-20-2588 PMC8026569

[26] Hagiwara M , Fushimi A, Yamashita N, Battacharya A, Rajabi H, Long M, . MUC1-C activates the PBAF chromatin remodeling complex in integrating redox balance with progression of human prostate cancer stem cells. Oncogene 2021;40:4930–40. 34163028 10.1038/s41388-021-01899-y PMC8321896

[27] Yamashita N , Morimoto Y, Fushimi A, Ahmad R, Bhattacharya A, Daimon T, . MUC1-C dictates PBRM1-mediated chronic induction of interferon signaling, DNA damage resistance and immunosuppression in triple-negative breast cancer. Mol Cancer Res 2023;21:274–89.. 36445328 10.1158/1541-7786.MCR-22-0772 PMC9975675

[28] Bhattacharya A , Fushimi A, Yamashita N, Hagiwara M, Morimoto Y, Rajabi H, . MUC1-C dictates JUN and BAF-mediated chromatin remodeling at enhancer signatures in cancer stem cells. Mol Cancer Res 2022;20:556–67. 35022313 10.1158/1541-7786.MCR-21-0672 PMC8983489

[29] Bhattacharya A , Fushimi A, Wang K, Yamashita N, Morimoto Y, Ishikawa S, . MUC1-C intersects chronic inflammation with epigenetic reprogramming by regulating the SET1A compass complex in cancer progression. Commun Biol 2023;6:1030. 37821650 10.1038/s42003-023-05395-9 PMC10567710

[30] Moses MA , George AL, Sakakibara N, Mahmood K, Ponnamperuma RM, King KE, . Molecular mechanisms of p63-mediated squamous cancer pathogenesis. Int J Mol Sci 2019;20:3590. 31340447 10.3390/ijms20143590 PMC6678256

[31] Pecorari R , Bernassola F, Melino G, Candi E. Distinct interactors define the p63 transcriptional signature in epithelial development or cancer. Biochem J 2022;479:1375–92. 35748701 10.1042/BCJ20210737 PMC9250260

[32] Fisher ML , Balinth S, Mills AA. DeltaNp63alpha in cancer: importance and therapeutic opportunities. Trends Cell Biol 2023;33:280–92. 36115734 10.1016/j.tcb.2022.08.003 PMC10011024

[33] Boumahdi S , Driessens G, Lapouge G, Rorive S, Nassar D, Le Mercier M, . SOX2 controls tumour initiation and cancer stem-cell functions in squamous-cell carcinoma. Nature 2014;511:246–50. 24909994 10.1038/nature13305

[34] Kalafut J , Czerwonka A, Anameric A, Przybyszewska-Podstawka A, Misiorek JO, Rivero-Muller A, . Shooting at moving and hidden targets-tumour cell plasticity and the Notch signalling pathway in head and neck squamous cell carcinomas. Cancers 2021;13:6219. 34944837 10.3390/cancers13246219 PMC8699303

[35] Kondratyev M , Pesic A, Ketela T, Stickle N, Beswick C, Shalev Z, . Identification of acquired Notch3 dependency in metastatic head and neck cancer. Commun Biol 2023;6:538. 37202533 10.1038/s42003-023-04828-9 PMC10195806

[36] Morimoto Y , Yamashita N, Daimon T, Hirose H, Yamano S, Haratake N, . MUC1-C is a master regulator of MICA/B NKG2D ligand and exosome secretion in human cancer cells. J Immunother Cancer 2023;11:e006238. 36754452 10.1136/jitc-2022-006238 PMC9923360

[37] Charan J , Kantharia ND. How to calculate sample size in animal studies? J Pharmacol Pharmacother 2013;4:303–6. 24250214 10.4103/0976-500X.119726 PMC3826013

[38] Choi JH , Lee BS, Jang JY, Lee YS, Kim HJ, Roh J, . Single-cell transcriptome profiling of the stepwise progression of head and neck cancer. Nat Commun 2023;14:1055. 36828832 10.1038/s41467-023-36691-x PMC9958029

[39] Khodarev N , Ahmad R, Rajabi H, Pitroda S, Kufe T, McClary C, . Cooperativity of the MUC1 oncoprotein and STAT1 pathway in poor prognosis human breast cancer. Oncogene 2010;29:920–9. 19915608 10.1038/onc.2009.391 PMC2820589

[40] Haratake N , Ozawa H, Morimoto Y, Yamashita N, Daimon T, Bhattacharya A, . MUC1-C is a common driver of acquired osimertinib resistance in non-small cell lung cancer. J Thorac Oncol 2024;19:434–50. 37924972 10.1016/j.jtho.2023.10.017 PMC10939926

[41] Khodarev NN . Intracellular RNA sensing in mammalian cells: role in stress response and cancer therapies. Int Rev Cell Mol Biol 2019;344:31–89. 30798990 10.1016/bs.ircmb.2018.08.005

[42] Li T , Chen ZJ. The cGAS-cGAMP-STING pathway connects DNA damage to inflammation, senescence, and cancer. J Exp Med 2018;215:1287–99. 29622565 10.1084/jem.20180139 PMC5940270

[43] Mazewski C , Perez RE, Fish EN, Platanias LC. Type I interferon (IFN)-regulated activation of canonical and non-canonical signaling pathways. Front Immunol 2020;11:606456. 33329603 10.3389/fimmu.2020.606456 PMC7719805

[44] Michalska A , Blaszczyk K, Wesoly J, Bluyssen HAR. A positive feedback amplifier circuit that regulates interferon (IFN)-stimulated gene expression and controls type I and type II IFN responses. Front Immunol 2018;9:1135. 29892288 10.3389/fimmu.2018.01135 PMC5985295

[45] Weichselbaum RR , Ishwaran H, Yoon T, Nuyten DS, Baker SW, Khodarev N, . An interferon-related gene signature for DNA damage resistance is a predictive marker for chemotherapy and radiation for breast cancer. Proc Natl Acad Sci U S A 2008;105:18490–5. 19001271 10.1073/pnas.0809242105 PMC2587578

[46] Khodarev NN , Roizman B, Weichselbaum RR. Molecular pathways: interferon/stat1 pathway: role in the tumor resistance to genotoxic stress and aggressive growth. Clin Cancer Res 2012;18:3015–21. 22615451 10.1158/1078-0432.CCR-11-3225

[47] Benci JL , Xu B, Qiu Y, Wu TJ, Dada H, Twyman-Saint Victor C, . Tumor interferon signaling regulates a multigenic resistance program to immune checkpoint blockade. Cell 2016;167:1540–54. 27912061 10.1016/j.cell.2016.11.022 PMC5385895

[48] Sandy Z , da Costa IC, Schmidt CK. More than meets the ISG15: emerging roles in the DNA damage response and beyond. Biomolecules 2020;10:1557. 33203188 10.3390/biom10111557 PMC7698331

[49] Qiu J , Xu B, Ye D, Ren D, Wang S, Benci JL, . Cancer cells resistant to immune checkpoint blockade acquire interferon-associated epigenetic memory to sustain T cell dysfunction. Nat Cancer 2023;4:43–61. 36646856 10.1038/s43018-022-00490-y

[50] Wardlaw CP , Petrini JHJ. ISG15: a link between innate immune signaling, DNA replication, and genome stability. Bioessays 2023;45:e2300042. 37147792 10.1002/bies.202300042 PMC10473822

[51] Honkala AT , Tailor D, Malhotra SV. Guanylate-binding protein 1: an emerging target in inflammation and cancer. Front Immunol 2020;10:3139. 32117203 10.3389/fimmu.2019.03139 PMC7025589

[52] Wu ZH , Cai F, Zhong Y. Comprehensive analysis of the expression and prognosis for GBPs in head and neck squamous cell carcinoma. Sci Rep 2020;10:6085. 32269280 10.1038/s41598-020-63246-7 PMC7142114

[53] Prendergast GC , Malachowski WP, DuHadaway JB, Muller AJ. Discovery of IDO1 inhibitors: from bench to bedside. Cancer Res 2017;77:6795–811. 29247038 10.1158/0008-5472.CAN-17-2285 PMC6021761

[54] Adam I , Dewi DL, Mooiweer J, Sadik A, Mohapatra SR, Berdel B, . Upregulation of tryptophanyl-tRNA synthethase adapts human cancer cells to nutritional stress caused by tryptophan degradation. Oncoimmunology 2018;7:e1486353. 30524887 10.1080/2162402X.2018.1486353 PMC6279332

[55] Ahn YH , Oh SC, Zhou S, Kim TD. Tryptophanyl-tRNA synthetase as a potential therapeutic target. Int J Mol Sci 2021;22:4523. 33926067 10.3390/ijms22094523 PMC8123658

[56] Miranda A , Hamilton PT, Zhang AW, Pattnaik S, Becht E, Mezheyeuski A, . Cancer stemness, intratumoral heterogeneity, and immune response across cancers. Proc Natl Acad Sci U S A 2019;116:9020–9. 30996127 10.1073/pnas.1818210116 PMC6500180

[57] De Angelis ML , Francescangeli F, La Torre F, Zeuner A. Stem cell plasticity and dormancy in the development of cancer therapy resistance. Front Oncol 2019;9:626. 31355143 10.3389/fonc.2019.00626 PMC6636659

[58] Quintanal-Villalonga A , Chan JM, Yu HA, Pe'er D, Sawyers CL, Sen T, . Lineage plasticity in cancer: a shared pathway of therapeutic resistance. Nat Rev Clin Oncol 2020;17:360–71. 32152485 10.1038/s41571-020-0340-z PMC7397755

[59] Crum CP , McKeon FD. p63 in epithelial survival, germ cell surveillance, and neoplasia. Annu Rev Pathol 2010;5:349–71. 20078223 10.1146/annurev-pathol-121808-102117

[60] You WK , Schuetz TJ, Lee SH. Targeting the DLL/Notch signaling pathway in cancer: challenges and advances in clinical development. Mol Cancer Ther 2023;22:3–11. 36223541 10.1158/1535-7163.MCT-22-0243 PMC9808372

[61] Woo HG , Choi JH, Yoon S, Jee BA, Cho EJ, Lee JH, . Integrative analysis of genomic and epigenomic regulation of the transcriptome in liver cancer. Nat Commun 2017;8:839. 29018224 10.1038/s41467-017-00991-w PMC5635060

[62] Kufe D . Chronic activation of MUC1-C in wound repair promotes progression to cancer stem cells. J Cancer Metastasis Treat 2022;8:12. 35539431 10.20517/2394-4722.2022.03 PMC9083497

[63] Chakraborty R , Darido C, Liu F, Maselko M, Ranganathan S. Head and neck cancer Immunotherapy: molecular biological aspects of preclinical and clinical research. Cancers 2023;15:852. 36765809 10.3390/cancers15030852 PMC9913716

[64] Papalouka C , Adamaki M, Batsaki P, Zoumpourlis P, Tsintarakis A, Goulielmaki M, . DNA damage response mechanisms in head and neck cancer: significant implications for therapy and survival. Int J Mol Sci 2023;24:2760. 36769087 10.3390/ijms24032760 PMC9917521

[65] Yamashita N , Long M, Fushimi A, Yamamoto M, Hata T, Hagiwara M, . MUC1-C integrates activation of the IFN-gamma pathway with suppression of the tumor immune microenvironment in triple-negative breast cancer. J Immunother Cancer 2021;9:e002115. 33495298 10.1136/jitc-2020-002115 PMC7839859

[66] Yamashita N , Fushimi A, Morimoto Y, Bhattacharya A, Hagiwara M, Yamamoto M, . Targeting MUC1-C suppresses chronic activation of cytosolic nucleotide receptors and STING in triple-negative breast cancer. Cancers 2022;14:2580. 35681561 10.3390/cancers14112580 PMC9179855

[67] Dekoninck S , Blanpain C. Stem cell dynamics, migration and plasticity during wound healing. Nat Cell Biol 2019;21:18–24. 30602767 10.1038/s41556-018-0237-6 PMC7615151

[68] Ge Y , Gomez NC, Adam RC, Nikolova M, Yang H, Verma A, . Stem cell lineage infidelity drives wound repair and cancer. Cell 2017;169:636–50. 28434617 10.1016/j.cell.2017.03.042 PMC5510746

[69] Watanabe K , Villarreal-Ponce A, Sun P, Salmans ML, Fallahi M, Andersen B, . Mammary morphogenesis and regeneration require the inhibition of EMT at terminal end buds by Ovol2 transcriptional repressor. Dev Cell 2014;29:59–74. 24735879 10.1016/j.devcel.2014.03.006 PMC4062651

[70] Watanabe H , Ma Q, Peng S, Adelmant G, Swain D, Song W, . SOX2 and p63 colocalize at genetic loci in squamous cell carcinomas. J Clin Invest 2014;124:1636–45. 24590290 10.1172/JCI71545 PMC3973117

[71] Greten FR , Grivennikov SI. Inflammation and cancer: triggers, mechanisms, and consequences. Immunity 2019;51:27–41. 31315034 10.1016/j.immuni.2019.06.025 PMC6831096

