# Supplementary material for: Identification of MUC1-C as a Target for Suppressing Progression of Head and Neck Squamous Cell Carcinomas
Source: Cancer Res Commun. 2024 May 14;4(5):1268–81. doi: 10.1158/2767-9764.CRC-24-0011 (PMC11092937; doi:10.1158/2767-9764.CRC-24-0011)
Supplement: Figure S1 — Effects of silencing MUC1-C on HNSCC cell clonogenic survival. [file crc-24-0011-s01.docx]

**
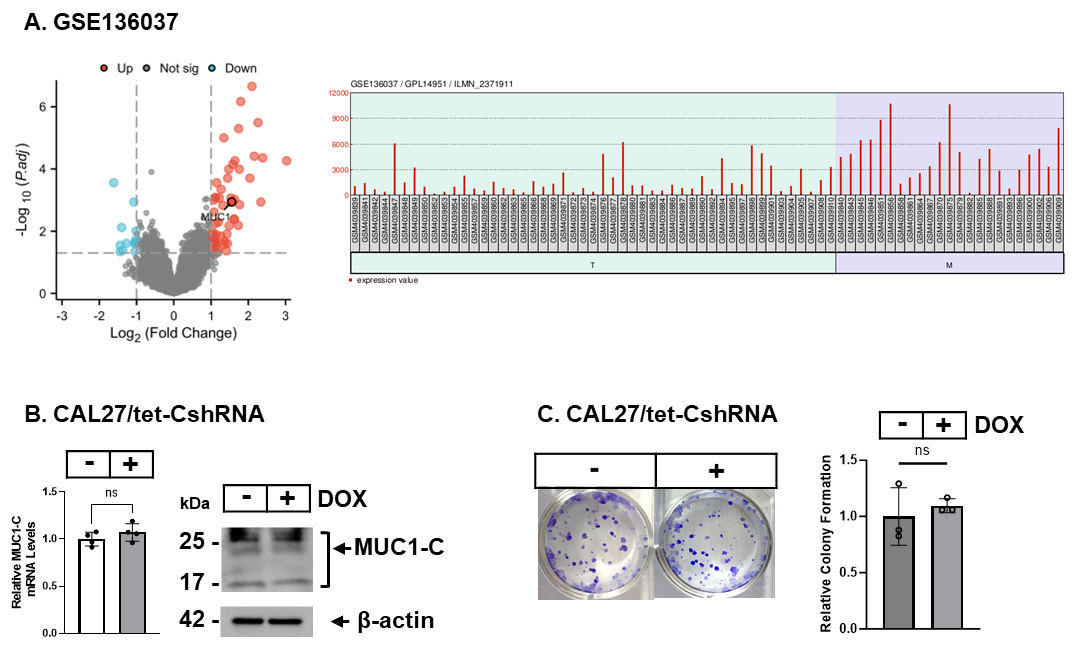
**

**Supplemental Figure S1. Effects of silencing MUC1-C on HNSCC cell clonogenic survival. A.** Volcano plot of down- and up-regulated genes in metastatic vs primary HNSCCs in the GSE136037 dataset (left). Levels of MUC1 expression in primary and metastatic HNSCCs (right). **B.** CAL27/tet-CshRNA cells treated with vehicle or DOX for 7 days were analyzed for MUC1-C mRNA levels (left). The results (mean±SD of four determinations) are expressed as relative levels compared to that obtained for vehicle-treated cells (assigned a value of 1) (left). Lysates were immunoblotted with antibodies against the indicated proteins (right). **C.** CAL27/tet-CshRNA cells treated with vehicle or DOX for 7 days were analyzed for colony formation. Shown are representative photomicrographs of stained colonies (left). The results (mean±SD of three determinations) are expressed as relative colony formation compared to that for vehicle-treated cells (assigned a value of 1)(right).
