# Supplementary material for: Identification of MUC1-C as a Target for Suppressing Progression of Head and Neck Squamous Cell Carcinomas
Source: Cancer Res Commun. 2024 May 14;4(5):1268–81. doi: 10.1158/2767-9764.CRC-24-0011 (PMC11092937; doi:10.1158/2767-9764.CRC-24-0011)
Supplement: Figure S2 — MUC1-C regulates the type I and IFN pathways in CAL27 and HSC3 cells. [file crc-24-0011-s02.docx]

**
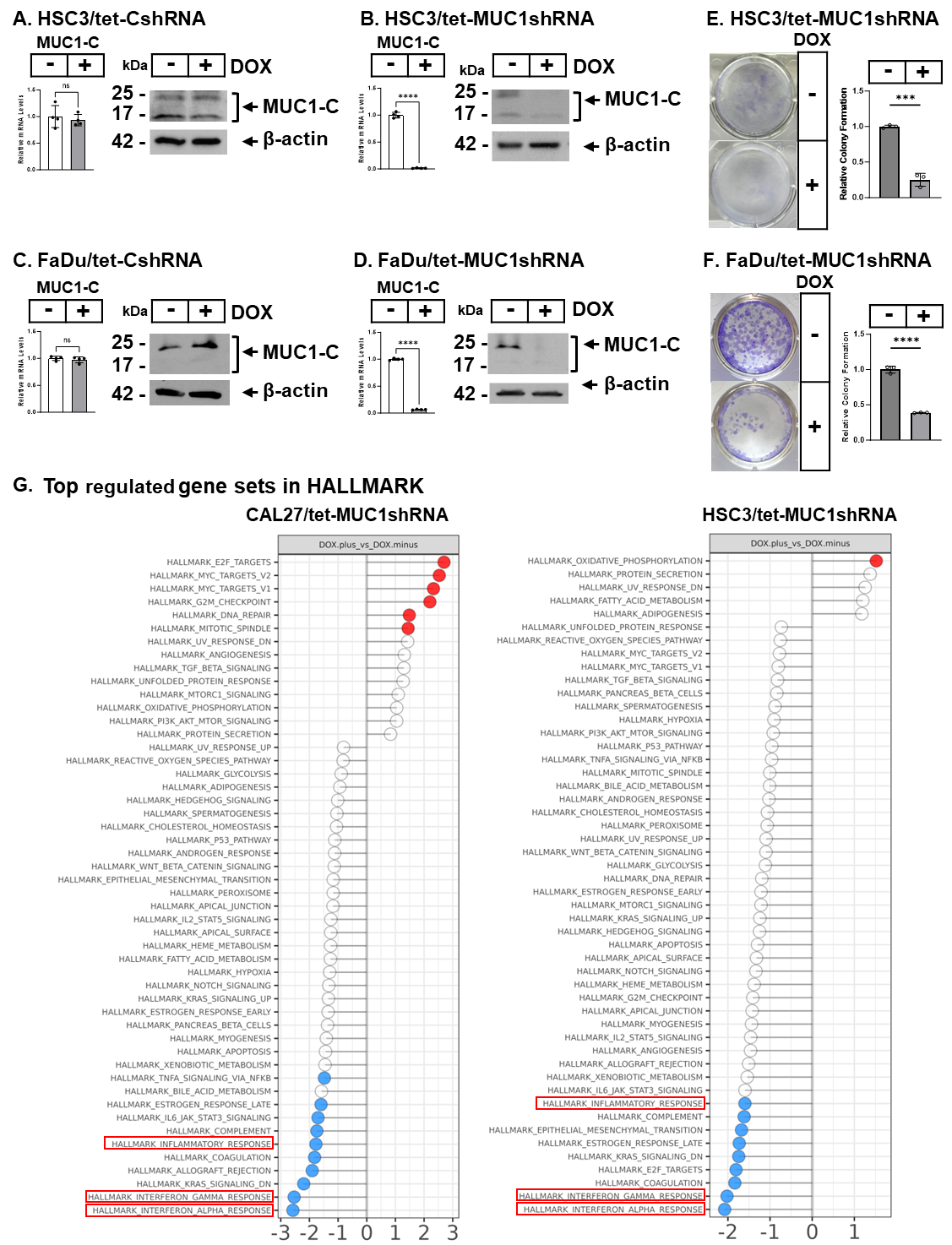
**

**Supplemental Fig. S2. MUC1-C regulates the type I and IFN pathways in CAL27 and HSC3 cells. A and B.** HSC3/tet-CshRNA (**A**) and HSC3/tet-MUC1shRNA (**B**) cells treated with vehicle or DOX for 7 days were analyzed for MUC1-C mRNA levels (left). The results (mean±SD of four determinations) are expressed as relative levels compared to that obtained for vehicle-treated cells (assigned a value of 1) (left). Lysates were immunoblotted with antibodies against the indicated proteins (right). **C and D.** FaDu/tet-CshRNA (**C**) and FaDu/tet-MUC1shRNA (**D**) cells treated with vehicle or DOX for 7 days were analyzed for MUC1-C mRNA levels (left). The results (mean±SD of four determinations) are expressed as relative levels compared to that obtained for vehicle-treated cells (assigned a value of 1) (left). Lysates were immunoblotted with antibodies against the indicated proteins (right). **E-F.** HSC3/tet-MUC1shRNA (**E**) and FaDu/tet-MUC1shRNA (**F**) cells treated with vehicle or DOX for 7 days were analyzed were analyzed for colony formation. Shown are representative photomicrographs of stained colonies (left). The results (mean±SD of three determinations) are expressed as relative colony formation compared to that for vehicle-treatedcells (assigned a value of 1)(right). **G.** Effects of silencing MUC1-C on the indicated HALLMARK gene signatures.
