# Supplementary material for: Identification of MUC1-C as a Target for Suppressing Progression of Head and Neck Squamous Cell Carcinomas
Source: Cancer Res Commun. 2024 May 14;4(5):1268–81. doi: 10.1158/2767-9764.CRC-24-0011 (PMC11092937; doi:10.1158/2767-9764.CRC-24-0011)
Supplement: Figure S3 — Effects of targeting MUC1-C on effectors of the type I and II IFN pathways. [file crc-24-0011-s03.docx]

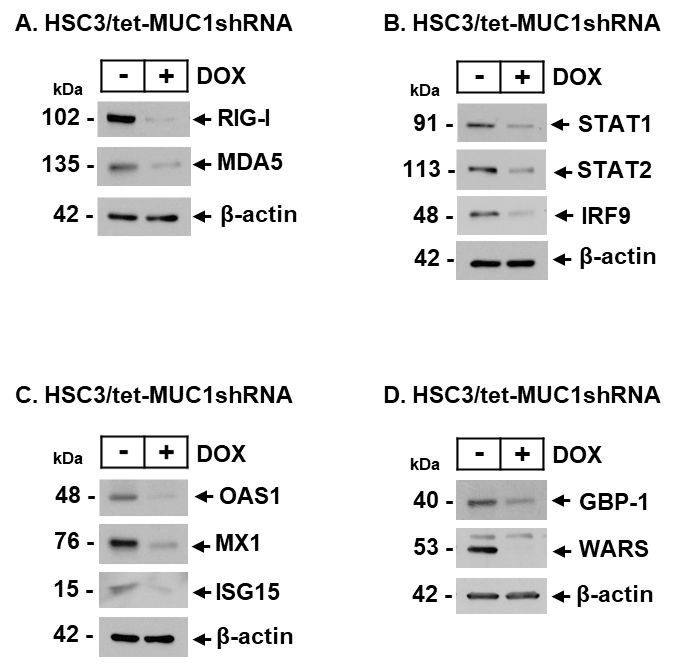


**Supplemental Fig. S3. Effects of targeting MUC1-C on effectors of the type I and II IFN pathways. A-D.** Lysates from HSC3/tet-MUC1shRNA cells treated with vehicle of DOX for 7 days were immunoblotted with antibodies against the indicated proteins.
