# Supplementary material for: Identification of MUC1-C as a Target for Suppressing Progression of Head and Neck Squamous Cell Carcinomas
Source: Cancer Res Commun. 2024 May 14;4(5):1268–81. doi: 10.1158/2767-9764.CRC-24-0011 (PMC11092937; doi:10.1158/2767-9764.CRC-24-0011)
Supplement: Figure S4 — Regulation of ∆Np63 expression in HSC3 cells. [file crc-24-0011-s04.docx]

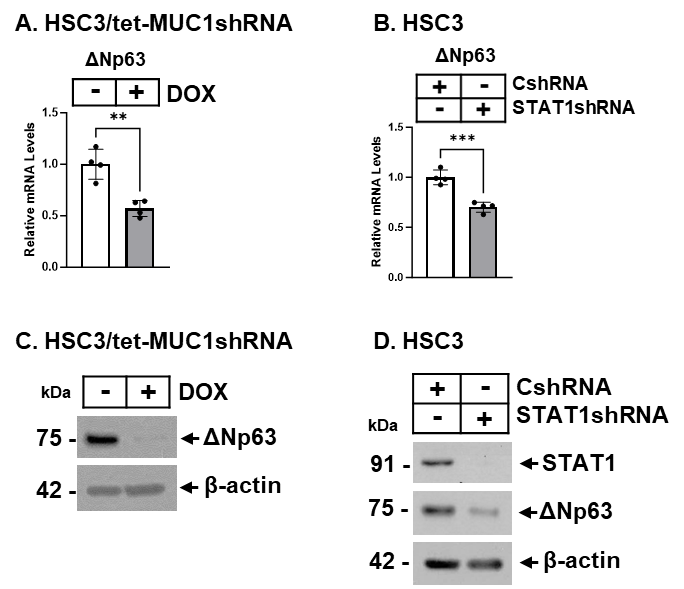


**Supplemental Figure S4. Regulation of ∆Np63 expression in HSC3 cells. A.** HSC3/tet-MUC1shRNA cells treated with vehicle or DOX for 7 days were analyzed for ∆Np63 mRNA levels. The results (mean±SD of four determinations) are expressed as relative levels compared to that obtained for vehicle-treated cells (assigned a value of 1). **B.** HSC3/CshRNA and CAL27/STAT1shRNA cells were analyzed for ∆Np63 mRNA levels. The results (mean±SD of four determinations) are expressed as relative levels compared to that obtained for vehicle-treated cells (assigned a value of 1). **C.** Lysates from HSC3/tet-MUC1shRNA cells treated with vehicle or DOX for 7 days were immunoblotted with antibodies against the indicated proteins. **D.** Lysates from HSC3/CshRNA and CAL27/STAT1shRNA cells were immunoblotted with antibodies against the indicated proteins.
