# Supplementary material for: Identification of MUC1-C as a Target for Suppressing Progression of Head and Neck Squamous Cell Carcinomas
Source: Cancer Res Commun. 2024 May 14;4(5):1268–81. doi: 10.1158/2767-9764.CRC-24-0011 (PMC11092937; doi:10.1158/2767-9764.CRC-24-0011)
Supplement: Figure S5 — Regulation of SOX2 expression in HSC3 cells. [file crc-24-0011-s05.docx]

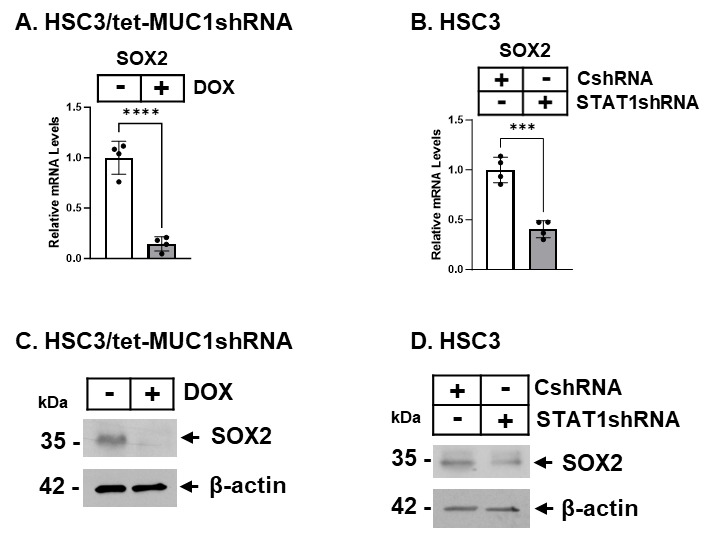


**Supplemental Figure S5. Regulation of SOX2 expression in HSC3 cells. A.** HSC3/tet-MUC1shRNA cells treated with vehicle or DOX for 10 days were analyzed for SOX2 mRNA levels. The results (mean±SD of four determinations) are expressed as relative levels compared to that obtained for vehicle-treated cells (assigned a value of 1). **B.** HSC3/CshRNA and HSC3/STAT1shRNA cells were analyzed for SOX2 mRNA levels. The results (mean±SD of four determinations) are expressed as relative levels compared to that obtained for vehicle-treated cells (assigned a value of 1).
