# Supplementary material for: Identification of MUC1-C as a Target for Suppressing Progression of Head and Neck Squamous Cell Carcinomas
Source: Cancer Res Commun. 2024 May 14;4(5):1268–81. doi: 10.1158/2767-9764.CRC-24-0011 (PMC11092937; doi:10.1158/2767-9764.CRC-24-0011)
Supplement: Figure S6 — Effects of targeting MUC1-C with the GO-203 inhibitor. [file crc-24-0011-s06.docx]

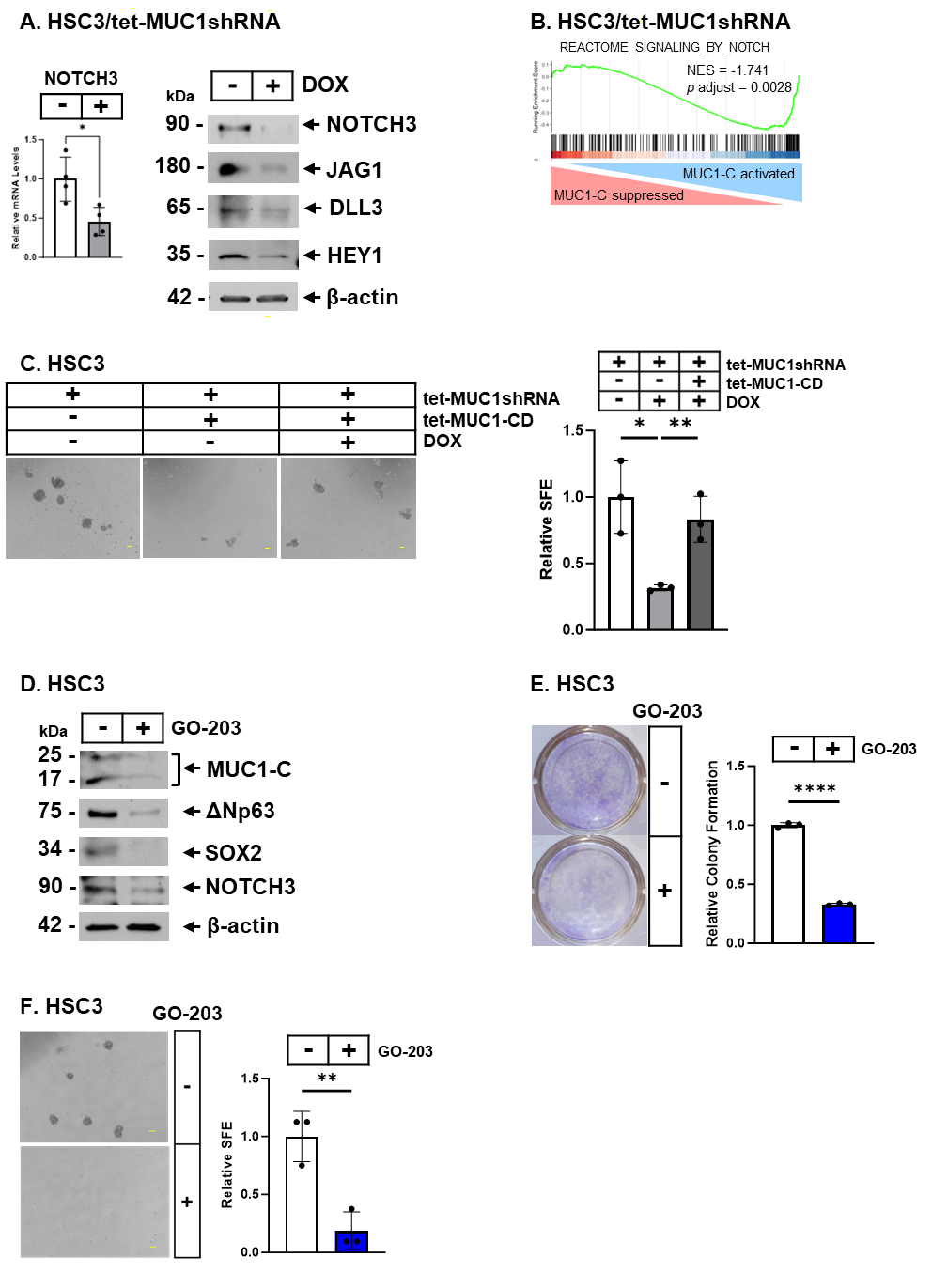


**Supplemental Figure S6. Effects of targeting MUC1-C with the GO-203 inhibitor. A.** CAL27/tet-MUC1shRNA cells treated with vehicle or DOX for 7 days were analyzed for NOTCH3 mRNA levels. The results (mean±SD of four determinations) are expressed as relative levels compared to that obtained for vehicle-treated cells (assigned a value of 1)(left). Lysates were immunoblotted with antibodies against the indicated proteins (right). **B.** GSEA of RNA-seq data from HSC3/tet-MUC1shRNA cells treated with vehicle or DOX for 7 days using the REACTOME SIGNALING BY NOTCH3 gene signature. **C.** The indicated HSC3 cells treated with vehicle or DOX for 7 days were analyzed for tumorsphere formation. Shown are representative images of the tumorspheres (bar represents 100 microns). Sphere forming efficiency (SFE) is expressed as the mean±SD of three independent replicates relative to that obtained for vehicle treated cells (assigned a value of 1). **D.** Lysates from HSC3 cells treated with vehicle or 5 μM GO-203 for 2 days were immunoblotted with antibodies against the indicated proteins. **E.** HSC3 cells treated with vehicle or 5 μM GO-203 were analyzed for colony formation. Shown are representative photomicrographs of stained colonies (left). The results (mean±SD of three determinations) are expressed as relative colony formation compared to that for vehicle treated cells (assigned a value of 1)(right). **F.** HSC3 cells were treated with vehicle or 5 μM GO-203 and were analyzed for tumorsphere formation. Shown are representative images of the tumorspheres (bar represents 100 microns). Sphere forming efficiency (SFE) is expressed as the mean±SD of three independent replicates relative to that obtained for untreated cells (assigned a value of 1).
