# Supplementary material for: Identification of MUC1-C as a Target for Suppressing Progression of Head and Neck Squamous Cell Carcinomas
Source: Cancer Res Commun. 2024 May 14;4(5):1268–81. doi: 10.1158/2767-9764.CRC-24-0011 (PMC11092937; doi:10.1158/2767-9764.CRC-24-0011)
Supplement: Figure S7 — Single-cell profiling of the expression of MUC1 and related genes in HNSCC. [file crc-24-0011-s07.docx]

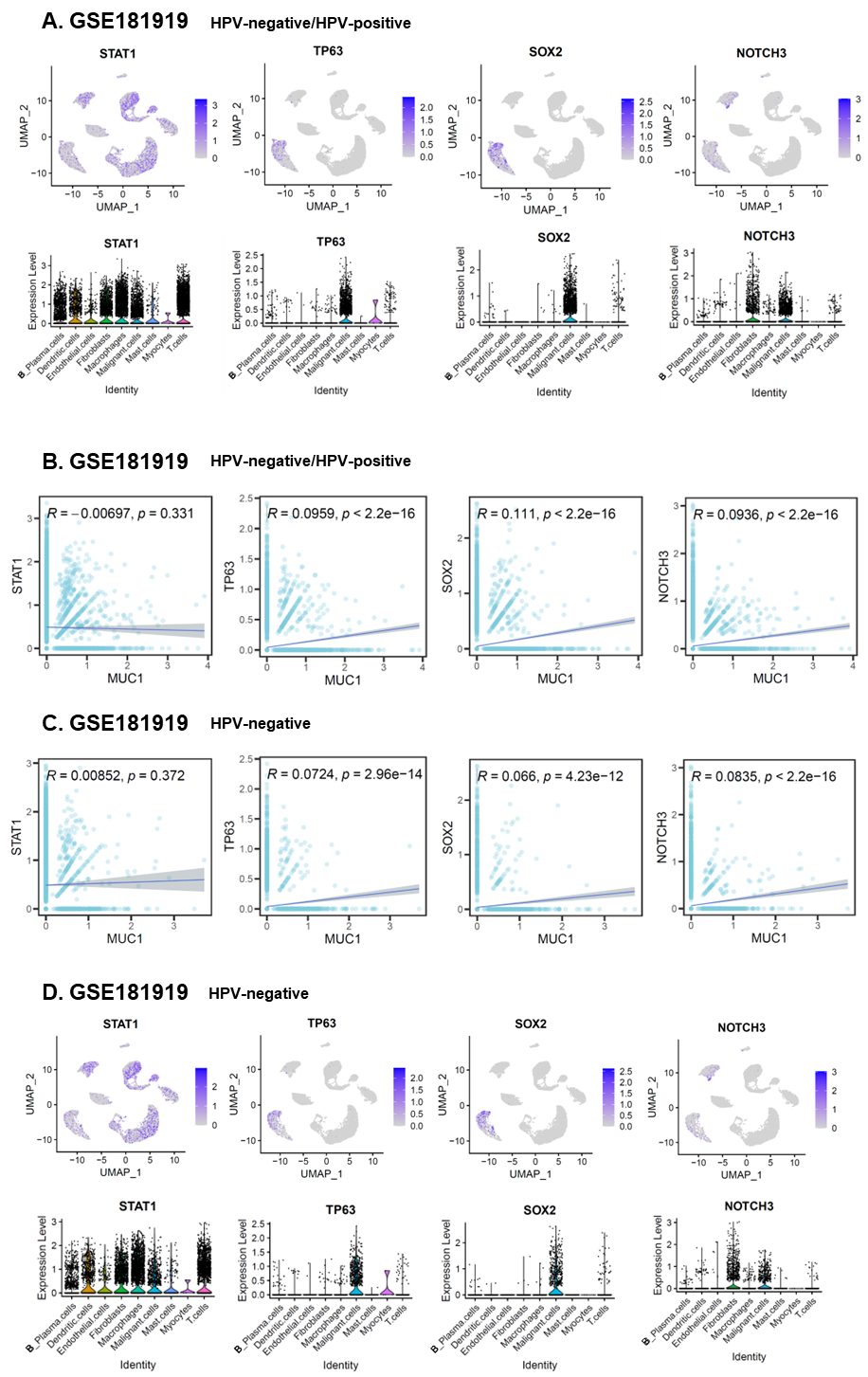


**Supplemental Figure S7. Single-cell profiling of the expression of MUC1 and related genes in HNSCC.** **A**. Expression of STAT1, TP63, SOX2 and NOTCH3 across UMAP projections (top) and in each cell type (below) in HPV-negative/HPV-positive HNSCC samples. **B and C**. Correlation analysis between MUC1 and STAT1, TP63, SOX2, NOTCH3 expression in identified malignant cells. **D.** Expression of STAT1, TP63, SOX2 and NOTCH3 across UMAP projections (top) and in each cell type (below).
