# Supplementary material for: Identification of MUC1-C as a Target for Suppressing Progression of Head and Neck Squamous Cell Carcinomas
Source: Cancer Res Commun. 2024 May 14;4(5):1268–81. doi: 10.1158/2767-9764.CRC-24-0011 (PMC11092937; doi:10.1158/2767-9764.CRC-24-0011)
Supplement: Table S1 — Primers used for qRT-PCR analysis. [file crc-24-0011-s08.docx]

**Supplemental Table S1. Primers used for qRT-PCR analysis.**

| **MUC1-C** | **FWD** | TACCGATCGTAGCCCCTATG |
| --- | --- | --- |
|  | **REV** | CTCACCAGCCCAAACAGG |
| **RIG-1** | **FWD** | CTGGACCCTACCTACATCCTG |
|  | **REV** | GGCATCCAAAAAGCCACGG |
| **MDA5** | **FWD** | CCATGGAGAAGGCTGGGG |
|  | **REV** | CAAAGTTGTCATGGATGACC |
| **STAT1** | **FWD** | GGAACTTGATGGCCCTAAAGGA |
|  | **REV** | ACAGAGCCCACTATCCGAGACA |
| **STAT2** | **FWD** | GCAGCACAATTTGCGGAA |
|  | **REV** | ACAGGTGTTTCGAGAACTGGC |
| **IRF9** | **FWD** | CCCGACCTCACCGATGAC |
|  | **REV** | TCTCGCGAAGCTGGATGTC |
| **OAS1** | **FWD** | TGAGGTCCAGGCTCCACGCT |
|  | **REV** | GCAGGTCGGTGCACTCCTCG |
| **MX1** | **FWD** | CTTTCCAGTCCAGCTCGGCA |
|  | **REV** | AGCTGCTGGCCGTACGTCTG |
| **ISG15** | **FWD** | CGCAGATCACCCAGAAGATCG |
|  | **REV** | TTCGTCGCATTTGTCCACCA |
| **GBP-1** | **FWD** | AGGAGTTCCTTCAAAGATGTGGA |
|  | **REV** | TTCTGAACAAAGAGACGATAGCC |
| **IDO-1** | **FWD** | TCTCATTTCGTGATGGAGACT |
|  | **REV** | GTGTCCCGTTCTTGCATTTGC |
| **WARS** | **FWD** | TGACGGATGACGAGAAGTATCT |
|  | **REV** | GCCGAAAATGCCTTTCACTTG |
| **ΔNp63** | **FWD** | GAAAACAATGCCCAGACTCAA |
|  | **REV** | TGCGCGTGGTCTGTGTTA |
| **SOX2** | **FWD** | GAGAGAAAGAAAGGGAGAGAAG |
|  | **REV** | GAGAGAGGCAAACTGGAATC |
| **NOTCH3** | **FWD** | TGGCGACCTCACTTACGACT |
|  | **REV** | CACTGGCAGTTATAGGTGTTGAC |
| **β-actin** | **FWD** | GATGAGATTGGCATGGCTTT |
|  | **REV** | CACCTTCACCGTTCCAGTTT |
