# Supplementary material for: Identification of MUC1-C as a Target for Suppressing Progression of Head and Neck Squamous Cell Carcinomas
Source: Cancer Res Commun. 2024 May 14;4(5):1268–81. doi: 10.1158/2767-9764.CRC-24-0011 (PMC11092937; doi:10.1158/2767-9764.CRC-24-0011)
Supplement: Table S2 — Primers used for ChIP-PCR. [file crc-24-0011-s09.docx]

**Supplemental Table S2. Primers used for ChIP-PCR.**

| **ΔNp63** | **FWD** | GATAACAGAACTCAAGTCCCTCTC |
| --- | --- | --- |
|  | **REV** | AGGTGGAAGTTGATGGATTGG |
| **SOX2** | **FWD** | GTCCCATCCTCATTTAAGTACCC |
|  | **REV** | CTTTGTATCCCCTCTCGCAG |
